# Bispecific T cell engager (BiTE^®^) antibody constructs can mediate bystander tumor cell killing

**DOI:** 10.1371/journal.pone.0183390

**Published:** 2017-08-24

**Authors:** Sandra L. Ross, Marika Sherman, Patricia L. McElroy, Julie A. Lofgren, Gordon Moody, Patrick A. Baeuerle, Angela Coxon, Tara Arvedson

**Affiliations:** 1 Department of Oncology Research, Amgen Inc., Thousand Oaks, California, United States of America; 2 Department of Amgen Research Munich, Amgen Inc., Munich, Germany; Mie University Graduate School of Medicine, JAPAN

## Abstract

For targets that are homogenously expressed, such as CD19 on cells of the B lymphocyte lineage, immunotherapies can be highly effective. Targeting CD19 with blinatumomab, a CD19/CD3 bispecific antibody construct (BiTE^®^), or with chimeric antigen receptor T cells (CAR-T) has shown great promise for treating certain CD19-positive hematological malignancies. In contrast, solid tumors with heterogeneous expression of the tumor-associated antigen (TAA) may present a challenge for targeted therapies. To prevent escape of TAA-negative cancer cells, immunotherapies with a local bystander effect would be beneficial. As a model to investigate BiTE^®^-mediated bystander killing in the solid tumor setting, we used epidermal growth factor receptor (EGFR) as a target. We measured lysis of EGFR-negative populations in vitro and in vivo when co-cultured with EGFR-positive cells, human T cells and an EGFR/CD3 BiTE^®^ antibody construct. Bystander EGFR-negative cells were efficiently lysed by BiTE^®^-activated T cells only when proximal to EGFR-positive cells. Our mechanistic analysis suggests that cytokines released by BiTE^®^-activated T-cells induced upregulation of ICAM-1 and FAS on EGFR-negative bystander cells, contributing to T cell-induced bystander cell lysis.

## Introduction

Recent clinical advances have demonstrated robust therapeutic activity of T cells in the treatment of patients with refractory or relapsed (r/r) acute B-lymphocytic leukemia (B-ALL), and with late-stage melanoma, bladder, head and neck, and non-small cell lung cancer. Blockade of the PD-L1/PD1 axis or of CTLA4 by respective monoclonal antibodies, or adoptive transfer of ex-vivo expanded tumor-infiltrating T cells, can unleash T cell activity and show strong therapeutic effects, including partial and complete responses and long-term remission in a fraction of patients. Targeted engagement of T cells using bispecific antibody constructs or chimeric antigen receptors (CARs) that directly connect T cells with tumor-associated surface antigens (TAAs) is another strategy. High response rates have been observed in patients with CD19-expressing r/r ALL by either approach [1]. Blinatumomab, a CD19/CD3-bispecific bispecific T cell engager (BiTE^®^) antibody construct, is the first molecule of this class approved in the U.S. and E.U. It induces durable complete remission in patients with minimal residual disease and r/r B-ALL [2, 3], and was shown to be effective in patients with non-Hodgkin’s lymphoma [4]. Additional BiTE^®^ antibody constructs are currently being evaluated in other hematological malignancies [3] and in solid tumor indications [2, 3, 5].

BiTE^®^ antibody constructs comprise tandemly-arranged single-chain variable fragments (scFvs). One scFv binds the TCR CD3ε subunit and the other binds a tumor-associated surface antigen (TAA). BiTE^®^ antibody constructs have been shown to induce the formation of a cytolytic synapse between the T cell and the transiently-linked tumor cell [6]. Target cell lysis occurs in the absence of regular major histocompatibility complex (MHC) class I/peptide antigen recognition and costimulation, and is therefore resistant to certain immune escape mechanisms affecting antigen presentation and those affecting generation of tumor-specific T cell clones [6, 7]. T cell activation by BiTE^®^ antibody constructs is strictly dependent on the presence of cells expressing the TAA. Because the CD3ε target of BiTE^®^ antibody construct is invariant, both CD8^+^ and CD4^+^ T cells of any phenotype can be engaged, leading to a polyclonal T cell activation, expansion and tumor cell lysis [7].

CD8^+^ and CD4^+^ effector T cells primarily use two pathways to kill target cells. The dominant mechanism involves the release of the secretory granule content of T cells; the secondary mechanism is more delayed compared to granule exocytosis and involves stimulation of death receptors on target cells by death receptor ligands on T cells [8–10]. The granule-mediated pathway requires formation of a cytolytic synapse induced by TCR/MHC I/peptide interaction. This synapse is stabilized by T cell-expressed lymphocyte function-associated antigen-1 (LFA-1) binding to intercellular adhesion molecule 1 (ICAM-1) on target cells. Once the synapse is formed, cytolytic proteins, including granzymes and perforin, are exocytosed by T cells. These cytolytic proteins form pores in target cells and elicit apoptosis [11]. In the death receptor pathway, TCR engagement induces expression of TNF superfamily ligands on the T cell surface that bind to and crosslink cell surface death receptors expressed on target cells [12]. Both pathways induce caspase-driven apoptosis of the target cell [12, 13].

One potential challenge for BiTE^®^ antibody construct therapeutics is that heterogeneity of TAA expression becomes a potential source of resistance, as treatment may only eliminate TAA-expressing tumor cells. This is also a concern for other targeted therapeutics, such as antibody drug conjugates, which are reported to only kill cells that have both high TAA expression and the ability to internalize and process the antibody-target complexes [14, 15]. A treatment modality with the potential to kill both TAA-positive tumor cells and proximal TAA-negative cells (bystanders) would be desirable to prevent escape and outgrowth of TAA-negative tumor cells during treatment. In solid tumors, TAA-negative cells include not only cancer cells but also stromal cells such as endothelial cells, tumor-associated macrophages (TAMs), or cancer-associated fibroblasts (CAFs). Tumor stroma can enhance tumorigenicity and metastasis [16, 17]. For complete tumor eradication, a bystander effect may therefore be preferred.

**Fig 1:**
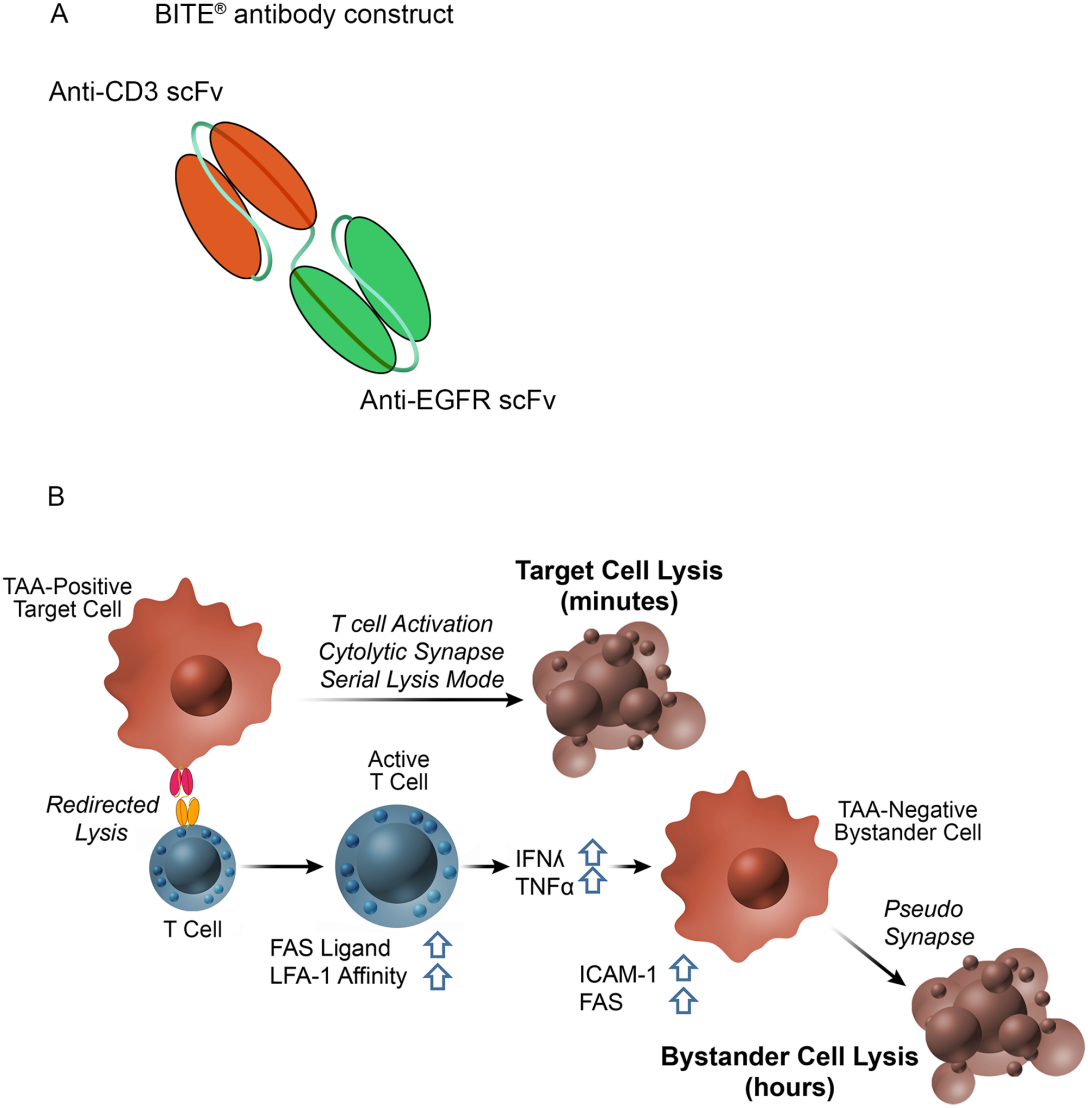
BiTE^®^ antibody constructs target both CD3Ԑ and a tumor-associated antigen and potential mechanism of bystander killing. (A) BiTE^®^ molecules are composed of an anti-CD3 scFv linked to an anti-tumor scFv; in this study, EGFR is used as the tumor antigen. BiTE^®^ molecules simultaneously engage the tumor associated antigen (TAA) on the tumor cell and CD3 on the T cell, resulting in T cell activation and T-cell mediated lysis of the tumor cell. (B) Model for bystander killing: T cells are activated by TAA-positive cells and BiTE^®^, resulting in formation of a cytolytic synapse and rapid lysis of target-positive cells. Activated T cells upregulate FASL, express high-affinity LFA-1 and release cytokines. IFNγ and TNFα secreted by activated T cells act on nearby TAA-negative bystander cells, inducing upregulation of cell-surface ICAM-1 and FAS. These molecules engage LFA-1 and FASL, respectively, on BiTE^®^-activated T cells. Expression of ICAM-1 and FAS, and likely other molecules, render bystander TAA-negative cells susceptible to killing by activated T cells. Unlike target-positive cell lysis, which occurs within minutes, bystander lysis requires several hours.

We provide evidence for bystander killing of EGFR-negative cells in vitro and in vivo using an EGFR-binding BiTE^®^ antibody construct (Fig 1A, [18]) and mixtures of EGFR-positive and -negative (bystander) cells co-cultured with human T cells. We show that IFNγ and TNFα are secreted by BiTE^®^-activated T cells but have no direct cytotoxic effect. Exposure of bystander cells to BiTE^®^-activated T cells induced the expression of the death receptor FAS as well as ICAM-1, a molecule involved in stabilizing cytolytic T cell synapses. ICAM-1 and FAS upregulation on bystander cells was also observed following treatment with recombinant IFNγ and TNFα. Blocking either ICAM-1 or FAS with neutralizing antibodies partially protected bystander cells from BiTE^®^-mediated T cell killing. These findings suggest that BiTE^®^-mediated bystander killing is cytokine-driven and may depend, at least partially, on expression of ICAM-1 and FAS on target cells (Fig 1B).

## Materials and methods

DX.DOI.ORG/10.17504/PROTOCOLS.IO.IB8CARW

### Cell lines

The cell lines NUGC4 [19], HCT116 [20], SW620 [21] and MOLM13 [22] were originally obtained from commercial sources and archived in an Amgen cell bank. Cell lines used in this study were authenticated by STR analysis at ATCC in July 2016. Cells were maintained in RPMI medium with 10% heat-inactivated FBS, 2mM glutamine and 1% penicillin/streptomycin at 37°C in 5% CO_2_.

### BiTE^®^ antibody constructs

The EGFR BiTE^®^ used in this study is based on cetuximab (Erbitux^®^) and has been previously described [18]. The MEC14 control BiTE^®^ is directed against an irrelevant herbicide antigen was previously described [23]. Both BiTE^®^ constructs recognize the same CD3Ԑ epitope.

### Antibodies

#### Immunofluorescence antibodies

Anti-human EGFR mouse monoclonal antibody (clone 199.12, ThermoFisher) was used at 1 μg/ml; anti-human ICAM-1 (CD54) mouse monoclonal antibody (clone MEM-111, Abcam) was used at 5 μg/ml; anti-human FAS (CD95) mouse monoclonal antibody (clone DX2, ThermoFisher) was used at 5 μg/ml.

#### Neutralizing antibodies

All antibodies used in functional assays were azide-free and previously shown to have neutralizing activity. Anti-human ICAM-1 mouse monoclonal antibody (clone 84H10, Beckman Coulter) was used at 5 μg/ml; anti-human FAS mouse monoclonal antibody (clone ZB4, Enzo) was used at 2.5 μg/ml; anti-human IFNγ R1 (CD119) mouse monoclonal antibodies (clones GIR208 and 92101, R&D Systems) and anti-human TNFRSF1A mouse monoclonal antibody (clone 16803, R&D Systems) were used at 2 μg/ml.

#### Activating antibody

Azide-free mouse monoclonal anti-human FAS activating (IgM) antibody (clone CH11, Millipore) was used at 2.5 μg/ml.

### T cells

Pan-T cells were purchased from AllCells. T cells that were not stimulated prior to use in assays are referred to as resting T cells. Activated T cells were generated by two methods: (1) for in vivo studies, activated T cells were generated using beads coated with anti-CD2, -CD28 and -CD3 antibodies for 3–7 days following the manufacturer’s instructions (Miltenyi Biotec T cell activation/expansion kit); (2) for in vitro assays, BiTE^®^-activated T cells were generated in bulk by culturing pan-T cells with NUGC4 cells (10:1) and 100 pM EGFR BiTE^®^ for 24 hours prior to use in assays. T cells were washed with fresh media before using in assays. Activation was confirmed using anti-CD25 and anti-CD69 antibodies (BD).

### T cell dependent cellular cytotoxicity (TDCC) assay

Cells were plated at 10,000 total cells/well in black Packard ViewPlate-96 plates (Perkin Elmer). Pan-T cells were added along with titrated EGFR BiTE^®^. Control wells contained no BiTE^®^ (with and without T cells). At the end of 48 hours, cytotoxicity was measured by nuclear count using cellular imaging. After washing, cells were fixed in 3.7% formaldehyde and stained with Hoechst nuclear dye (Invitrogen). Nuclei were counted in 9–16 10X fields from 2–6 replicate wells on a Thermo ArrayScan^™^ VTI using a size threshold in the nuclear channel to exclude any remaining T cells. For some assays, cytotoxicity was measured with CellTiter-Glo^®^ (Promega) or both cellular imaging and CellTiter-Glo^®^. Cytotoxicity EC_50_ values between the two methods were in good agreement. In some assays where multiple parameters were measured at the same time, replicates were limited to duplicate plates or duplicate wells to ensure assay quality and feasibility. In these large imaging data sets, each data point represented means for hundreds to thousands of individual cells, duplicates showed good precision, and dose response data demonstrated changes associated with the quantity of applied BiTE^®^.

### Mixed culture TDCC assay

NUGC4 (EGFR-positive) and SW620 (EGFR-negative) cells were mixed in various ratios and treated as described above. Fixed cells were stained with anti-EGFR (ThermoFisher) and Hoechst dye. An intensity threshold in the EGFR channel was used to classify cells as EGFR-positive or EGFR-negative; nuclei were counted as described above. Control wells with NUGC4 and SW620 cells alone were used to confirm the accuracy of the population analysis.

### T cell activation assay

T cells were removed from 96-well TDCC assays, centrifuged, washed and stained with CD45-APC, CD69-FITC and CD25-PE (BD Biosciences) for 30 min at 4C, followed by two washes. Events were acquired on FACS CANTO and analyzed with FCS Express. Four replicate wells from 96-well plates were combined to ensure sufficient events for the assay.

### Soluble factors

Neat or diluted cell supernatants were assayed according to manufacturer’s instructions (Meso Scale Discovery for IFNγ, TNFα, IL-1β and IL-6, RayBiotech ELISA for FAS ligand and Abcam for Granzyme B (CTLA-1). In some cases, medium from replicate wells were combined to ensure sufficient material for the assay.

### Animal care

Animal experiments were executed in strict compliance with institutional guidelines and regulations. Research and technical procedures performed on animals under this study (protocol #2008–00094) were approved by the IACUC (Institutional Animal Care and Use Committee for Amgen). Animals were housed in an Association for the Assessment and Accreditation of Laboratory Animal Care accredited facility and were cared for according to standards established in the Guide for the Care and Use of Laboratory Animals, 8^th^ edition, written by a subcommittee of the National Research Council at the Institute for Laboratory Animal Research. Animals were purchased from Charles River Laboratories (Wilimington, MA). During the experiment, animals were monitored at least once daily, and twice when indicated. A protocol was in place for monitoring animals and initiating humane endpoints when needed. The clinical signs used to determine when to euthanize if necessary included: failing to eat or drink; failing to make normal postural adjustments; failing to ambulate or bear weight on one or more limbs; exhibiting excoriation, mutilation, obvious distress, cachexia, respiratory distress; having >25% body weight loss after cytotoxic dosing or >20% body weight loss unrelated to cytotoxic dosing, having excessive tumor burden, or loss of >15% of initial body weight and exhibiting any additional signs of toxicity including inactivity, hunched posture or depression; failure to regain 85% of initial body weight by the next dosing cycle; severe eye injury (rupture or proptosis); having a tumor exceeding 20% of body weight or openly ulcerated and wet tumors. Methods to alleviate suffering included: (1) limiting the length of studies; (2) avoiding death as an endpoint; (3) use of aseptic surgical procedures; (4) administering appropriate anesthetic and analgesic agents to minimize discomfort; (5) limiting tumor growth to ≤ 1 cm; (6) monitoring animals closely to avoid ongoing discomfort; (7) group housing with species-appropriate enrichment and (8) ad lib access to food and water. None of the animals died prior to the experimental endpoint. At the end of the study, animals were euthanized by CO_2_ overdose, followed by a secondary physical method.

### In vivo assay

Female athymic nude mice (Charles River Laboratories) were used at approximately seven weeks old for this study. EGFR-positive and -negative cells were implanted at 250,000 and 500,000 cells per animal. Equal numbers (250,000 cells each) of luciferase-labeled EGFR-negative cells (SW620-LUC, see Methods) were mixed with EGFR-positive cells (HCT116) and bead-activated human T-cells (1:1), then subcutaneously implanted into athymic nude mice. MEC14 control or EGFR BiTE^®^ was dosed (0.05mg/kg) intraperitoneally once daily starting one day after tumor cell implantation (day 1) and continued until day 20; the study was terminated on day 21. Animals were anesthetized with isoflurane (inhalation to effect) prior to measurement of luminescence with an IVIS imaging system two times/week. Tumor volume was measured using calipers on days 8, 11, 15 and 21. Body weight was measured each time tumor volume was measured.

### Supernatant transfer and Transwell^®^ assays

Titrated EGFR BiTE^®^ and pan-T cells (10:1) were added to NUGC4 cells in 96-well plates and incubated for 48 hours. Supernatants from these plates were either transferred directly or clarified by centrifugation prior to transfer to 96-well plates containing SW620 cells that had been plated 5 hours previously. The plates were further incubated for 48 hours. As a control, SW620 cells were cultured with T cells and EGFR BiTE^®^ for 48 hours. Cytotoxicity was measured by nuclear count with cellular imaging. To determine which filter size would exclude T cells, HTS Transwell-96^®^ System (Corning) assays were set up with 1 μm and 5 μm membranes. T cells alone or T cells with NUGC4 cells + EGFR BiTE^®^ were added to the top chamber and incubated for 48 hours. T cell migration through the membrane was measured by assaying the bottom chamber with CellTiter-Glo^®^ (Promega). Subsequently NUGC4 cells and T cells +/- EGFR BiTE^®^ were added to the top chamber and SW620 cells were added to the bottom chamber and incubated for 48 hours. SW620 cell cytotoxicity was measured by assaying the bottom chamber with CellTiter-Glo^®^.

### ICAM-1 and FAS indirect immunofluorescence

Target cells were plated at 10,000 cells/well in Packard ViewPlate-96 and incubated 18–24 hours with and without the addition of IFNγ and TNFα (Roche) or resting or BiTE^®^-activated T cells. Cells were fixed in 3.7% formaldehyde (ThermoFisher) and stained with anti-ICAM (Abcam) or anti-FAS (ThermoFisher) and Hoechst nuclear dye (ThermoFisher). Goat anti-mouse Alexa Fluor^®^ 488 or 647 secondary antibodies (ThermoFisher) were used for detection. Untreated cells were used to set fluorescence thresholds for percent positive.

### Blocking assays

For ICAM-1 and FAS assays, SW620 cells were plated at 10,000 total cells/well in black Packard ViewPlate-96 plates and treated with 10ng/ml IFNγ and 5 ng/ml TNFα. After 18–24 hours, media were removed and a 2X solution of anti-ICAM-1 (clone 84H10, Beckman Coulter), anti-FAS (clone ZB4, Enzo) or mouse IgG1 control (Abcam) antibody was added to wells. The plate was then incubated for 60 minutes at 37°C prior to addition of resting or BiTE^®^-activated T cells (10:1 E:T ratio) and incubation for 24 hours. FAS activating antibody (clone CH11, Millipore) was used as a control for FAS activation. Cytotoxicity (nuclear count) was measured by cellular imaging as described. For IFNγR1, TNFRSF1A blocking assays, SW620 cells were treated as described above, except that there was no cytokine pretreatment. Anti-IFNγ R1 (clones GIR208 and 92101, R&D Systems) and anti-TNFRSF1A (clone 16803, R&D Systems) were used as neutralizing antibodies.

### Luciferase (LUC) labeling

Parental SW620 and MOLM-13 cells were transduced with lentivirus expressing Firefly Luciferase under the huEF1-α promoter at MOI of 5. The vector carried the blasticidin resistance gene and transduction was followed by blasticidin selection. After 3–4 weeks of cell passages, the Luc-labelled cell lines were tested by p24 ELISA to confirm RCL (Replication Competent Lentivirus) negativity.

### EGFR antibody and BiTE^®^ binding

SW620 cells and NUGC4 cells were incubated for 30 minutes on ice with titrated cetuximab C225 antibody (0. 02-20ug/ml), EGFR or MEC14 BiTE^®^ (0. 02–1000 nM) in FACS buffer (PBS/2% FBS), washed twice with ice-cold FACS buffer and incubated with detection antibodies (goat anti-human-AlexaFluor 488 for cmab and anti-his-Alexa Fluor^®^ 647 for BiTE^®^s) for 30 minutes on ice, followed by two washes. Cells were resuspended in FACS buffer containing propidium iodide to exclude dead cells and analyzed on a CANTO flow cytometer.

### Calculations, graphs and statistic

Cytotoxicity was measured by cellular imaging (nuclear counts) or by luminescence. Percent specific cytotoxicity = 100*[1-(BiTE^®^-treated units/no BiTE^®^ control units)], where units = luminescence units (Steady-Glo^®^ or CellTiter-Glo^®^, Promega). GraphPad Prism^®^ 6.07 was used for graph generation and analysis. Four-parameter variable slope nonlinear regression was used for dose response curve fitting. Statistical significance was determined using an unpaired t test and two-tailed P values; for unequal variances, Welch’s correction was used.

## Results

### EGFR BiTE^®^ induced T cell activation, cytokine release, FASL expression and target cell lysis in the presence of EGFR-expressing cells

In our studies, we used EGFR-positive and -negative human gastrointestinal cancer cell lines, and an EGFR/CD3-bispecific BiTE^®^ antibody construct [18]. NUGC4 cells expressed moderate levels and SW620 cells expressed undetectable levels of EGFR mRNA, as determined by RNA sequencing (Table 1) and EGFR cell-surface protein, as determined by live cell flow cytometry (S1 Fig).

**Table 1:** EGFR mRNA expression in cell lines used in this study.

Cell Line	Tissue Type	FPKM	EGFR Status
NUGC4	gastric cancer	12.73	positive
SW620	colon cancer	0.08	negative
HCT116	colon cancer	16.71	positive
MOLM13	acute myelogenous leukemia	0.06	negative

EGFR expression levels derived from the Cancer Cell Line Encyclopedia (CCLE) database were determined by RNA sequencing as fragments per kilobase of exon per million fragments mapped (FPKM). A value of <0.1 FPKM is considered negative.

**Fig 2:**
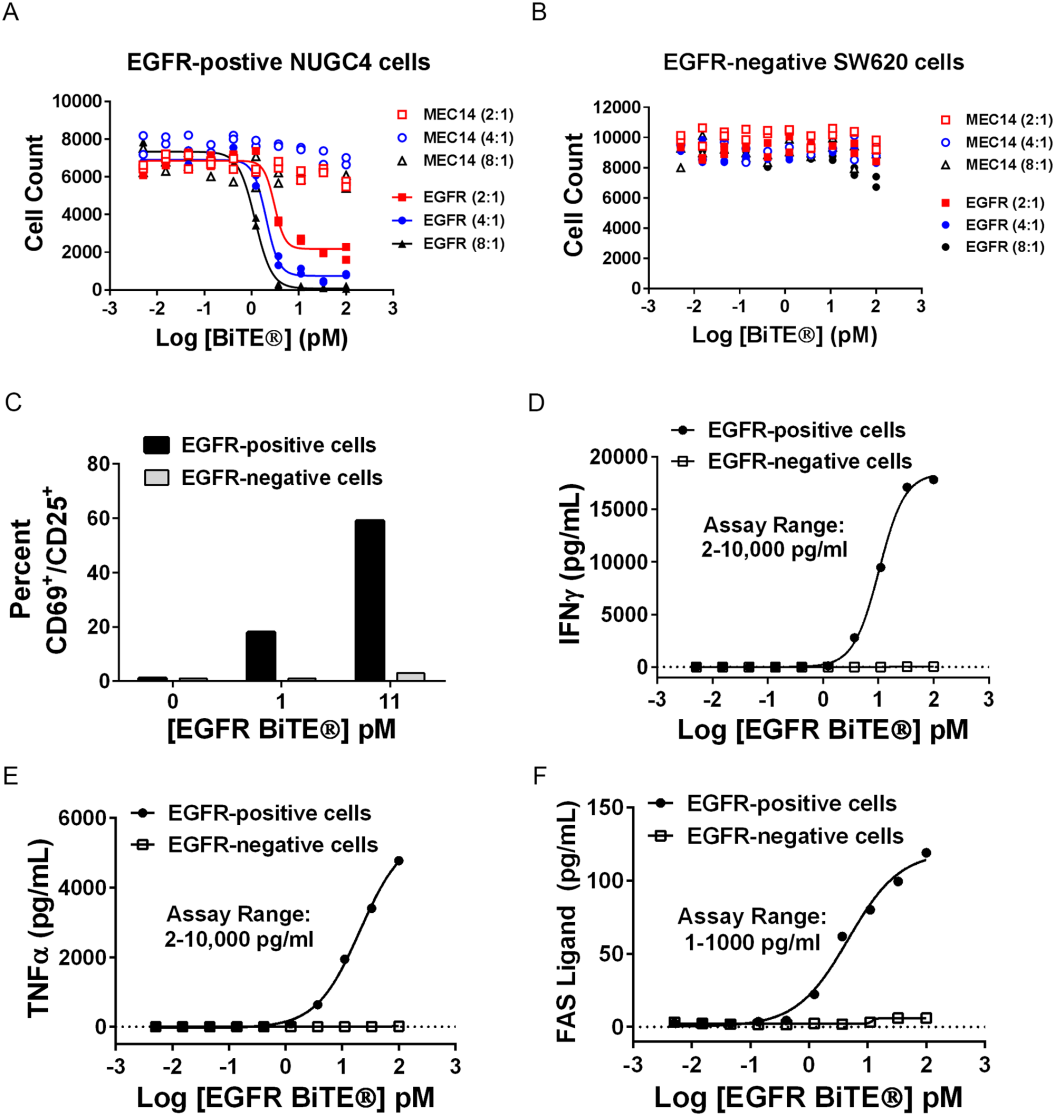
T cells were activated by EGFR BiTE^®^ in the presence of EGFR-expressing cells. (A) NUGC4 (EGFR-positive) or (B) SW620 (EGFR-negative) cells were incubated for 40 hours with EGFR BiTE^®^ or MEC14 negative control BiTE^®^ at E:T ratios of 2:1, 4:1 and 8:1. Cytotoxicity was measured by nuclear count with cellular imaging (N = 2, all data points shown). (C-F) Target cells, T cells (E:T ratio 7:1) and EGFR BiTE^®^ were incubated for 48 hours. Supernatants from 4 replicate wells were combined for each data point prior to separating T cells and media. (C) Percent CD69+/CD25+ cells was determined by flow cytometry. (D-F) Cytokine concentrations were determined by commercially available ELISA or MSD assays. Data shown for T cell activation and cytokine release are representative assays that were repeated at least twice.

In experiments using EGFR-positive NUGC4 cells and purified human T cells, EGFR BiTE^®^-activated T cells mediated highly potent redirected lysis with EC_50_ values between 1 and 10 pM EGFR BiTE^®^ antibody. Lysis was dependent on BiTE^®^ concentration and the ratio of T effector cells to target cells (E:T ratio) (Fig 2A). In contrast, EGFR-negative SW620 cells were not susceptible to BiTE^®^-mediated killing by T cells even at the highest BiTE^®^ concentration of 100 pM and at the highest E:T ratio of 8:1 (Fig 2B). A control BiTE^®^ antibody construct, MEC14, directed against an irrelevant antigen, but using the same CD3 engager [23], did not mediate lysis of either the NUGC4 or SW620 cells (Fig 2A and 2B). The dependency of BiTE^®^-mediated cytotoxicity on target expression and on E:T ratio was very reproducible and has been well documented in the literature [24–27]. T cell activation (Fig 2C) and secretion of pro-inflammatory mediators including IFNγ (Fig 2D), TNFα (Fig 2E) and FAS ligand (Fig 2F) were only evident when T cells encountered EGFR-positive NUGC4 cells, not EGFR-negative SW620 cells. Similarly, secretion of IL-1β, IL-6 and granzyme B by T cells also increased with EGFR BiTE^®^ concentration only when T cells were combined with EGFR-positive cells (S1 Table). These results demonstrate that EGFR BiTE^®^-mediated cytotoxicity and T cell activation required the presence of TAA-expressing cells.

### EGFR-negative bystander cells became susceptible to lysis by BiTE^®^-activated T cells when co-cultured with EGFR-positive cells

**Fig 3:**
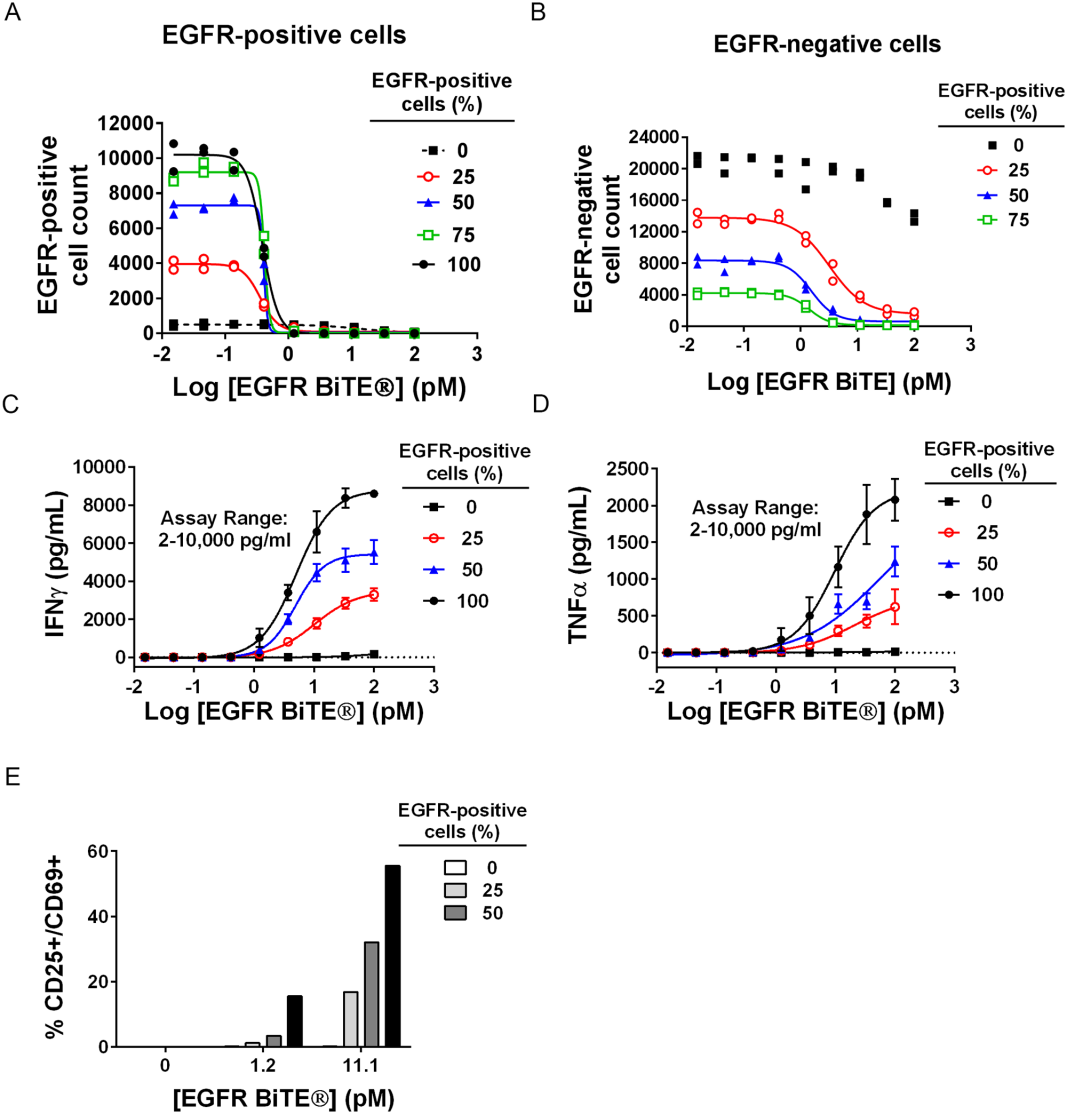
EGFR-negative bystander cells were lysed by BiTE^®^-activated T cells when co-cultured with EGFR-positive cells. NUGC4 (EGFR-positive) and SW620 (EGFR-negative) cells were mixed in various ratios and incubated with T cells (E:T ratio 10:1) and EGFR BiTE^®^ in duplicate plates. Thousands of cells/well were analyzed, with good agreement between replicate plates. This result was reproducible (S2C Fig). After 48 hours, cells were stained and analyzed as describe in Materials and Methods. Cytotoxicity of (A) EGFR-positive and (B) EGFR-negative cells was measured by nuclear count (N = 2, all data points shown). (C) IFNγ and (D) TNFα were measured using commercially available MSD assays (N = 3, mean +/- sd). (E) T cells from quadruplicate wells were combined and percent CD69+/CD25+ cells determined by flow cytometry.

To evaluate bystander killing, different ratios of EGFR-positive and -negative cells were mixed with human T cells and redirected lysis was determined over a wide range of EGFR BiTE^®^ concentrations. Cytotoxicity was quantified using an image-based assay in which EGFR-positive cells were identified using an anti-EGFR antibody (S2A and S2B Fig). EGFR-positive cells were lysed with an EC_50_ of 0.4 pM EGFR BiTE^®^ regardless of how many EGFR-negative cells were present (Fig 3A). EGFR-negative cells were also lysed, but this strictly depended on the presence of EGFR-expressing cells. A starting ratio of EGFR-positive to -negative cells of 1:3 was sufficient to induce robust lysis of EGFR-negative bystander cells with an EC_50_ of 3.2 pM EGFR BiTE^®^. With higher ratios of EGFR-positive cells, EC_50_ values for lysis of EGFR-negative cells were even lower at 1.5 pM (1:1 ratio) and 1.4 pM (3:1 ratio) (Fig 3B). Dependency of bystander killing on both BiTE^®^ concentration and ratio of target-positive cells was reproducible. A separate experiment with more replicates and fewer BiTE^®^ doses (S2C Fig) confirms EGFR-negative cells were susceptible to bystander killing that was dependent on BiTE^®^ dose and ratio of EGFR-positive cells present. Similar to the observed cytotoxicity, both cytokine release (IFNγ, Fig 3C and TNFα, Fig 3D), and T cell activation (Fig 3E), were detectable in cultures containing both EGFR-positive and -negative cells. The magnitude of the release and the degree of T cell activation were dependent on the ratio of EGFR-positive to -negative cells and the BiTE^®^ concentration.

To confirm that bystander killing was not unique to SW620 cells, a luciferase-expressing EGFR-negative acute myelogenous leukemia (AML) cell line, MOLM13-LUC (Table 1), was mixed with unlabeled EGFR-positive NUGC4 cells (1:1), T cells and EGFR BiTE^®^, and cytotoxicity was measured by luminescence. In this case, only the luciferase-labeled EGFR-negative MOLM13-LUC cells are measured. EGFR BiTE^®^ only induced lysis of the AML cell line when EGFR-positive cells were present; MOLM-13 cells were efficiently lysed, comparably to the AML positive control CD33 BiTE^®^ (S2D Fig). Thus, bystander killing can be observed in co-culture experiments with different TAA-negative cell lines.

### Lysis of bystander EGFR-negative tumor cells was observed in tumor xenografts of mice

**Fig 4:**
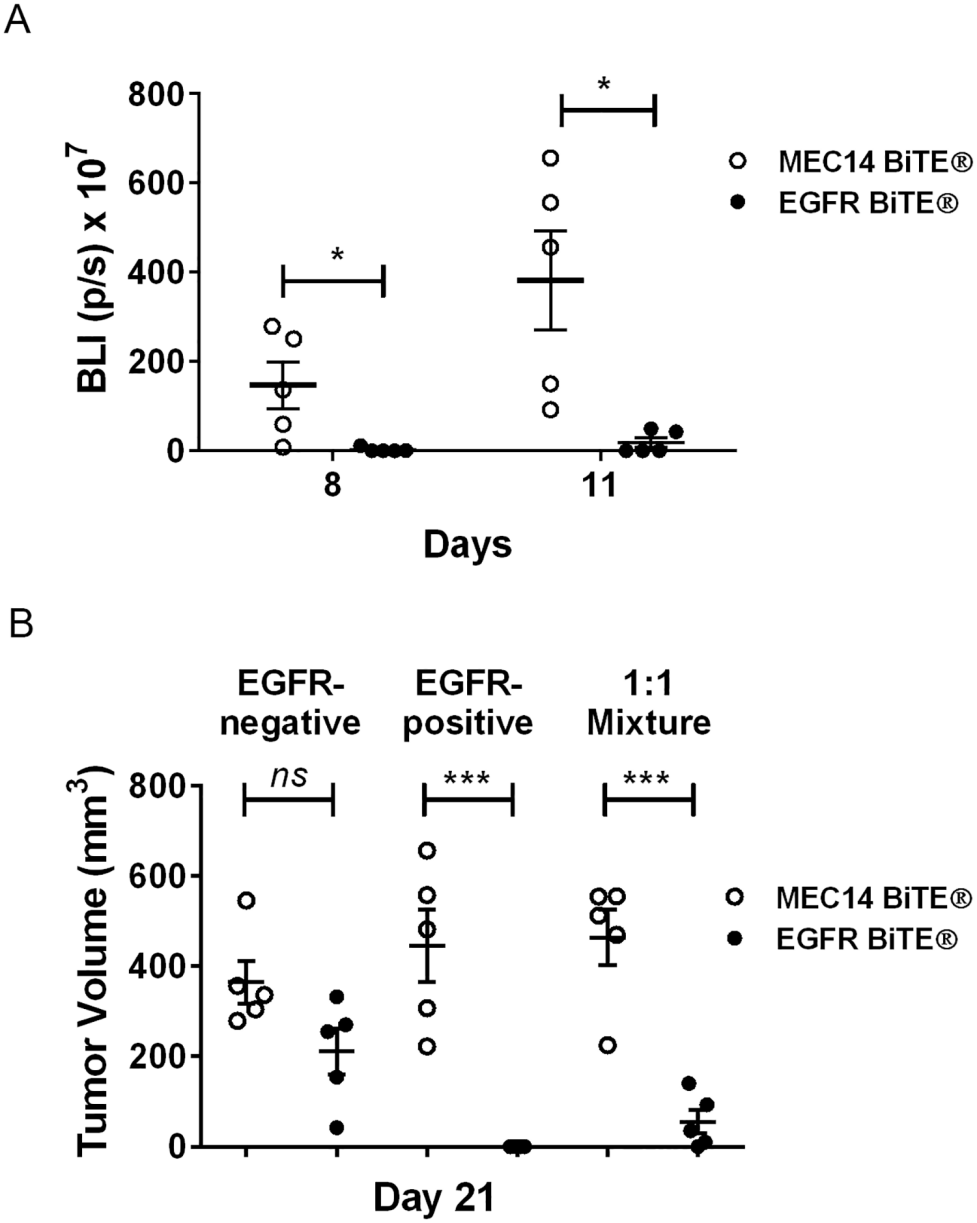
Lysis of bystander EGFR-negative tumor cells in tumor xenografts. Luciferase-labeled EGFR-negative cells (SW620-LUC), EGFR-positive cells (HCT116) or equal numbers of each cell line were mixed with human T cells (E:T 1:1, where the number of T cells is equal to the number of total combined target cells in mixed implants) and implanted in immunocompromised mice. MEC14 negative control BiTE^®^ or EGFR BiTE^®^ was dosed once daily. (A) Tumor growth for 1:1 mixture implants was measured by luminescence on days 8 and 11 using an imaging system. (B) Tumor volume was measured with calipers on day 21. Data represent averages of 5 replicate animals +/- SEM. Significance values: ns, P > 0.05; *P < 0.05; **P < 0.01; ***P < 0.001; ****P < 0.0001.

To investigate bystander killing in vivo, luciferase-labeled EGFR-negative SW620 cells (SW620-LUC) and EGFR-positive HCT116 cells (not luciferase-labeled), either alone or in a 1:1 ratio, were combined with human T cells (E:T 1:1, where target cell number in mixed cultures is the combined number of EGFR-positive and -negative cells) and implanted in immunocompromised mice. HCT116 cells were used because the growth rate was roughly equivalent to that of SW620 cells in vivo, and they have similar EGFR expression levels to NUGC4 (Table 1). EGFR BiTE^®^ EC_50_ values for in vitro lysis of HCT116 cells (0.7 pM) and NUGC4 cells (0.4 pM) were comparable (S3 Fig). Mice were treated daily with either the EGFR BiTE^®^ or the MEC14 negative control BiTE^®^ one day after tumor implantation. Despite inherent variability in xenograft growth in individual control animals, luminescence measurement at the midpoint (days 8, 11) of the study showed a significant reduction in luminesce of EGFR-negative SW620-LUC cells in the 1:1 mixed implants by EGFR BiTE^®^ treatment compared to treatment with the negative control BiTE^®^ (Fig 4A). Tumor volume at the end of the study (day 21) showed a highly significant growth inhibition with the EGFR BiTE^®^ vs. MEC14 control BiTE^®^ for implants with EGFR-positive cells or mixed EGFR-positive and -negative tumor cells, but not for implants containing only EGFR-negative tumor cells (Fig 4B). Small tumors that were histologically EGFR-negative and luciferase-positive were detected at the end of the study in some mice given mixed implants. There was no significant difference in tumor volume between the EGFR-positive and the 1:1 mixture groups treated with either EGFR BiTE^®^ or MEC14 control BiTE^®^. Thus, the bystander killing observed in vitro was recapitulated in a xenograft model.

### T cell-derived cytokines sensitized EGFR-negative cells for bystander killing

**Fig 5:**
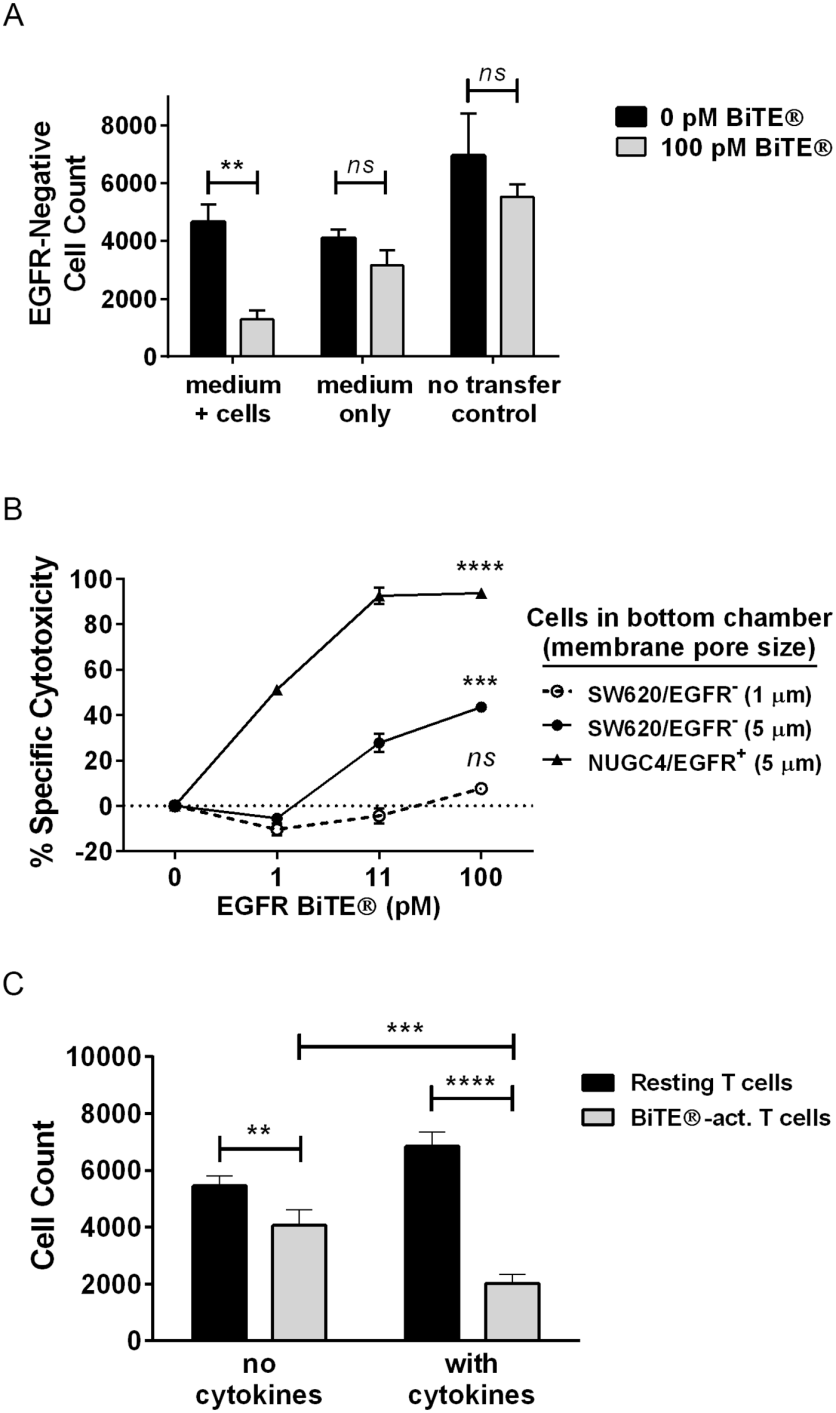
EGFR-negative cells were sensitized to bystander killing by T cell cytokines. (A) EGFR BiTE^®^, T cells and NUGC4 cells (E:T ratio 10:1) were incubated in 96-well plates for 48 hours; supernatants containing T cells were either transferred directly (medium + cells) or clarified by centrifugation prior to transfer (medium only) to 96-well plates containing SW620 cells, or SW620 cells were directly treated with T cells and EGFR BiTE^®^ (no transfer control); N = 3, mean +/- sd (B) T cells + EGFR BiTE^®^ + NUGC4 cells were added to the top chamber of Transwell^®^ assays with 1μm and 5μm membranes; SW620 (or NUGC4 as control) cells were added to the bottom chambers. Percent cytotoxicity in the bottom chambers was determined with CellTiter-Glo^®^. (C) SW620 cells were pre-treated for 24 hours +/- cytokines (10ng/ml IFNγ + 5ng/ml TNFα), then incubated for 24 hours with either resting T cells or BiTE^®^-activated T cells. Cells were enumerated by nuclear count with cellular imaging; N = 4, mean +/- sd. Significance values: ns, P > 0.05; *P < 0.05; **P < 0.01; ***P < 0.001; ****P < 0.0001.

To explore the mechanism of bystander killing, the effect of factors released by activated T cells was evaluated. T cells became activated and secreted multiple pro-inflammatory factors when incubated with T cells and EGFR BiTE^®^ (Figs 2 and 3, S1 Table, [18]). Crude supernatants containing medium and T cells, but not cell-free medium removed from such cultures induced significant cytotoxicity when transferred to EGFR-negative cells (Fig 5A; S4A and S4B Fig). As expected, no cytotoxicity was observed when EGFR-negative cells were treated directly with T cells plus EGFR BiTE^®^ (Fig 5A; S4C Fig). These results were corroborated with Transwell^®^ assays in which EGFR-positive cells, T cells and EGFR BiTE^®^ were placed in the top chamber, and EGFR-negative cells were placed in the bottom chamber; chambers were separated by membranes that limit transit of cells, but not soluble factors, between chambers. T cells can transit through a 5 μm, but not a 1 μm membrane, and have higher motility when activated by BiTE^®^ (S4D Fig). EGFR-negative cells in the bottom chamber were only killed when the membrane pore size was large enough to allow T cells to transit (Fig 5B). Thus, BiTE^®^-activated T cells in proximity to TAA-negative cells, rather than soluble factors, mediate bystander killing.

IFNγ and TNFα are produced at high levels by BiTE^®^-activated T cells (Fig 2, [28–30]), but clarified supernatants containing T cell-produced cytokines were not directly cytotoxic to SW620 cells. Likewise, exogenously added recombinant IFNγ and TNFα, alone or in combination, were not cytotoxic to the cell lines used in this study, even at concentrations exceeding those produced by BiTE^®^-activated T cells (S4E Fig). However, T cells activated by EGFR BiTE^®^ in the presence of EGFR-positive cells (i.e., BiTE^®^-activated T cells) lysed EGFR-negative cells. The degree of lysis was significantly higher when the EGFR-negative cells were pre-treated with IFNγ and TNFα (Fig 5C). These data indicate that these cytokines, while not directly cytotoxic, acted on EGFR-negative cells to increase their sensitivity to lysis by BiTE^®^-activated T cells.

### Bystander cells upregulated ICAM-1 and FAS in response to recombinant cytokines and in the presence of EGFR BiTE^®^-activated T cells

Pro-inflammatory cytokines such as IFNγ and TNFα can upregulate ICAM-1 (CD54) on multiple cell types [31]. ICAM-1 is a ligand for LFA-1 on lymphocytes and forms the peripheral supramolecular complex (pSMAC) of the immunological synapse. Upregulation of ICAM-1 in response to cytokines may therefore strengthen the transient interaction between effector and target cells [32].

**Fig 6:**
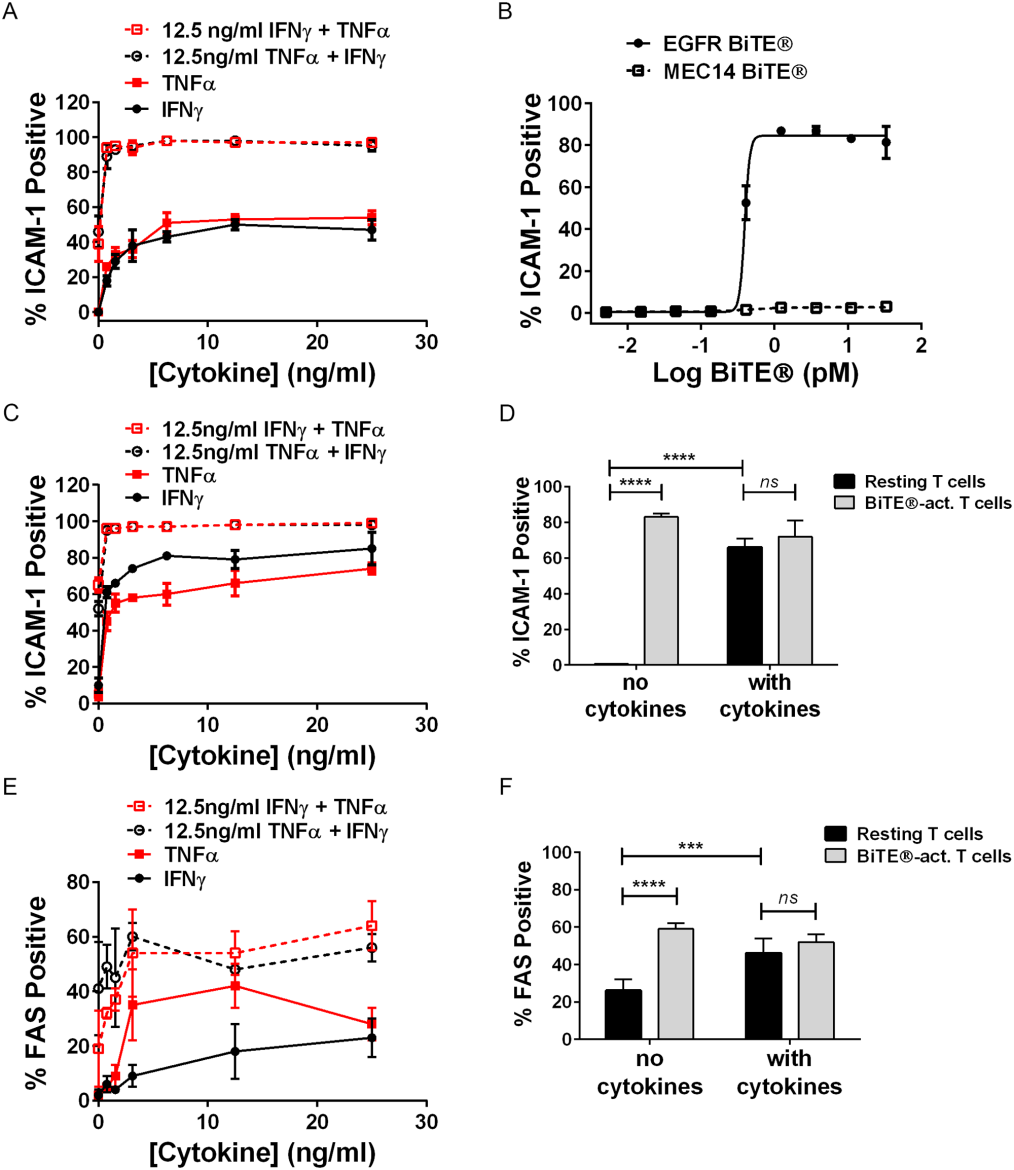
ICAM-1 and FAS were upregulated in response to recombinant cytokines or EGFR BiTE^®^-activated T cells. (A) NUGC-4 cells were treated with recombinant cytokines for 24 hours prior to staining with anti-ICAM-1 antibody; N = 3, mean +/- sd. (B) NUGC4 cells were treated with EGFR BiTE^®^ + T cells (E:T ratio 10:1) for 24 hours prior to staining with anti-ICAM-1 antibody; N = 3, mean +/- sd. (C) SW620 were treated with recombinant cytokines for 24 hours prior to staining with anti-ICAM-1 antibody; N = 3, mean +/- sd. (D) SW620 cells were pre-treated for 24 hours +/- cytokines (10ng/ml IFNγ + 5ng/ml TNFα), then incubated with either resting T cells or BiTE^®^-activated T cells for 24 hours prior to staining with anti-ICAM-1 antibody; N = 4, mean +/- sd. (E) SW620 cells were treated with recombinant cytokines for 24 hours prior to staining with anti-FAS antibody; N = 3, mean +/- sd. (F) SW620 cells were pre-treated for 24 hours +/- cytokines (10ng/ml IFNγ + 5ng/ml TNFα), then incubated with either resting T cells or BiTE^®^-activated T cells for 24 hours prior to staining with anti-FAS antibody; N = 6, mean +/- sd. Significance values: ns, P > 0.05; *P < 0.05; **P < 0.01; ***P < 0.001; ****P < 0.0001. Representative images shown in S5 Fig.

In our studies IFNγ and TNFα strongly induced ICAM-1 expression on EGFR-positive NUGC4 cells, especially when used in combination (Fig 6A, S5A Fig). The level of ICAM-1 induced by IFNγ and TNFα co-treatment was similar to that induced by BiTE^®^-activated T cells (Fig 6B, S5B Fig), and the degree of induction was dependent on EGFR BiTE^®^ concentration. ICAM-1 was similarly induced by recombinant cytokines in EGFR-negative SW620 cells (Fig 6C, S5C Fig), as well as by BiTE^®^-activated T cells (Fig 6D, S5D Fig); ICAM-1 induction by recombinant cytokines and BiTE^®^-activated T cells was comparable.

FAS ligand (FASL, CD95L) is expressed by cytotoxic T cells, natural killer cells [33], and by BiTE^®^-activated T cells (Fig 2, [30]). FAS (CD95) engagement is one mechanism by which immune cells kill virus-infected cells and tumor cells [34]. We found that FAS surface expression was upregulated in roughly half of the EGFR-negative SW620 cell population exposed to recombinant IFNγ and TNFα (Fig 6E, S5E Fig), or BiTE^®^-activated T cells (Fig 6F, S5F Fig).

### Blockade of IFNγ R1, TNFR1, ICAM-1 or FAS by neutralizing antibodies partially protected bystander cells from BiTE^®^-mediated cytotoxicity

**Fig 7:**
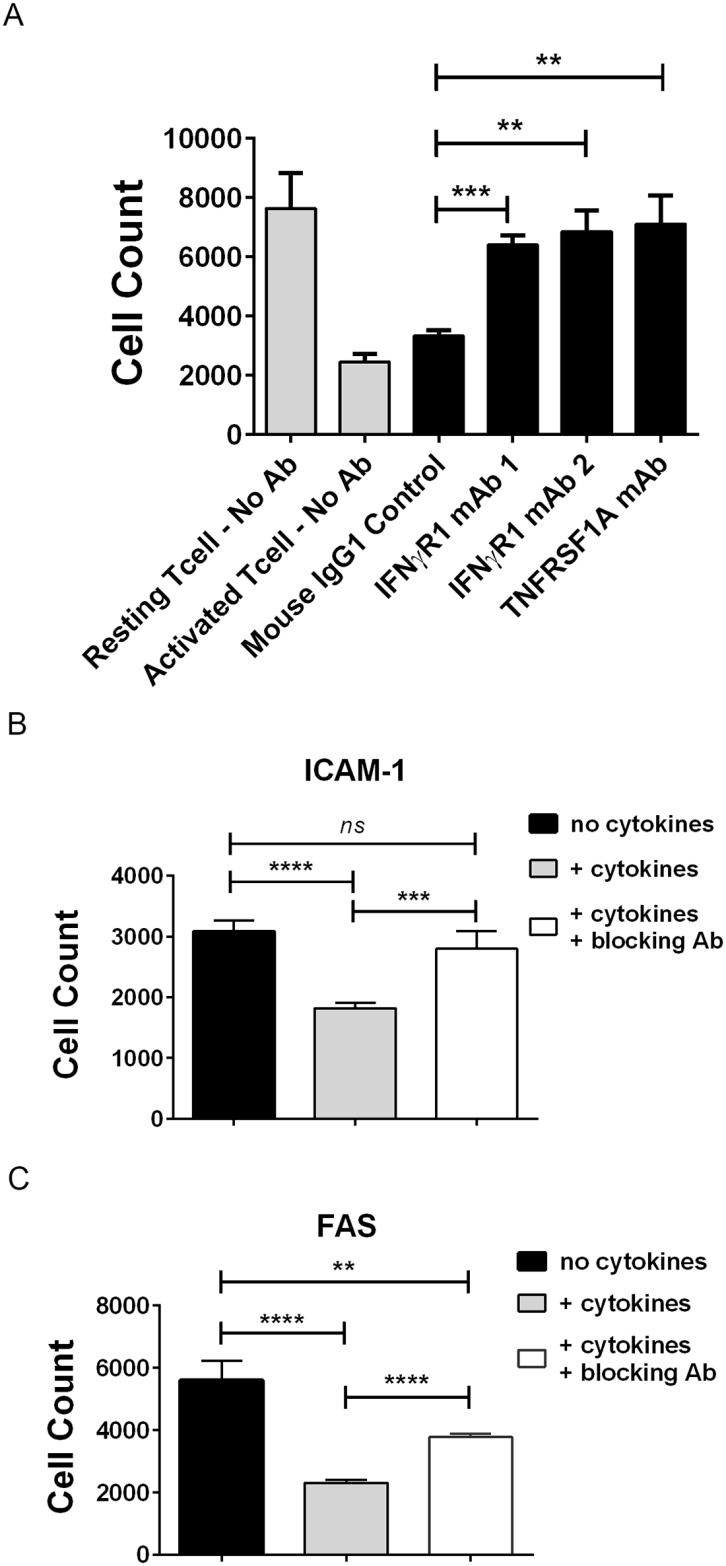
Blockade of IFNγ R1, TNFR1, ICAM-1 or FAS provided partial protection from BiTE^®^-mediated cytotoxicity. (A) SW620 cells were pretreated with either IFNγ R1- or TNFR1-blocking antibodies or mouse IgG1 control antibody at 2 μg/ml (final) for one hour prior to addition of resting T cells or BiTE^®^-activated T cells (E:T ratio 10:1) for 48 hours; N = 6, mean +/- sd. SW620 cells were pretreated with cytokines (5ng/ml IFNγ + 10ng/ml TNFα) for 18 hours to induce ICAM-1 and FAS, then incubated with (B) 5 μg/ml anti-ICAM-1 (final) or (C) 2.5 μg/ml anti-FAS (final) neutralizing antibodies (+ cytokines + blocking Ab) or control antibody (no cytokines and + cytokines) for one hour followed by addition of BiTE^®^-activated T cells (E:T ratio 10:1) for 24 hours. Cell count was determined by imaging; N = 4, mean +/- sd. Significance values: ns, P > 0.05; *P < 0.05; **P < 0.01; ***P < 0.001; ****P < 0.0001.

Pre-incubation of EGFR-negative SW620 cells with recombinant IFNγ and TNFα increased susceptibility to BiTE^®^-mediated bystander killing coinciding with an upregulation of ICAM-1 and FAS. To test whether ligand binding to IFNγ receptor (IFNγ R1), TNFα receptor (TNFRSF1A), ICAM-1 or FAS was involved in bystander killing, neutralizing antibodies were used to block these molecules on SW620 cells prior to incubating them with BiTE^®^-activated T cells. Blocking IFNγ R1 and TNFR1 significantly reduced bystander killing by BiTE^®^-activated T cells when compared to a control antibody (Fig 7A). Blocking either ICAM-1 (Fig 7B) or FAS (Fig 7C) on SW620 cells also had a significant and reproducible protective effect on BiTE^®^-mediated cytotoxicity, whereby the effect of neutralizing ICAM-1 was stronger than that of neutralizing FAS. As a control for FAS-mediated cytotoxicity, SW620 cells pretreated with cytokines to upregulate FAS were subsequently incubated with a FAS agonistic antibody, resulting in a degree of cytotoxicity that was completely abrogated by the FAS neutralizing antibody (S6 Fig, left panel). The FAS agonistic antibody had no effect on SW620 cells that had not been pretreated with cytokines (S6 Fig, right panel).

## Discussion

Obstacles for cancer therapies based on amplifying tumor-specific T cell responses include tumor heterogeneity and immune escape mechanisms, such as antigen loss, down-regulation of MHC class I, altered antigen processing, and an immunosuppressive tumor microenvironment. Bispecific CD3-engaging antibodies may have advantages over other T cell therapies because they can engage pre-existing polyclonal CD8- and CD4-positive T cells, do not require antigen presentation by MHC molecules, and bind to surface antigens on cancer cells that may be more homogenously expressed than MHC/peptide antigens. Nevertheless, the goal of all T cell therapies is tumor eradication, a task that is particularly challenging in solid tumors which are typically heterogeneous with respect to TAA expression. We anticipate that this will only be possible if both the TAA-expressing cancer cells and proximal TAA-negative cancer cells are eliminated by activated T cells. Bystander cells encompass TAA-negative cancer cells, endothelial cells, fibroblasts, macrophages and other immune cells. We focused our studies on bystander TAA-negative cancer cells as they may pose the greatest potential for resistance.

Previous reports evaluating BiTE^®^ antibody construct activity demonstrated that activation of T cells by BiTE^®^ is strictly dependent on target cell binding [24, 25, 29]; BiTE^®^ antibody constructs induce formation of functional cytolytic synapses [6] leading to apoptosis [35]; memory T cells are primarily responsible for redirected serial lysis [25] and BiTE^®^-mediated activation of T cells elicits release of multiple cytokines, including IL-2, IFNγ, TNFα, IL-6, IL-10 and IL-4 [28–30], which may have pleiotropic effects on nearby immune and non-immune cells. A variety of mouse models have shown that treatment with BiTE^®^ antibody constructs can eradicate established tumors with sizes up to 500 mm^3^ [29, 36].

Lysis of bystander TAA-negative cells has not been previously reported for BiTE^®^ antibody constructs. One study using a short-term microscopic assay to evaluate bystander killing reported no lysis of proximal target-negative cells [24]. Similar microscopic results were reported for ImmTACs, a BiTE^®^-like technology that targets MHC/peptide complexes [37]. We present here for the first time evidence that BiTE^®^-activated T cells can lyse non-TAA-expressing bystander cells when TAA-expressing target cells are present, and identify possible mechanisms for bystander killing. A starting ratio of one TAA-positive target cell to three TAA-negative bystander cells was sufficient to observe a complete bystander cell lysis of the co-culture at a low picomolar BiTE^®^ antibody construct concentration. Similarly, near-complete inhibition of xenograft outgrowth was observed in a mouse model, even when tumors initially contained 50 percent non-TAA-expressing cancer cells. These observations are seemingly in contrast to the notion that BiTE^®^-activated T cells lyse target cells by formation of a cytolytic immunological synapse formed between TAA-expressing cells and T cells. We therefore investigated in more detail the mode of bystander killing by BiTE^®^-activated T cells.

We could not detect induction of any EGFR target expression after treatment with either BiTE^®^ or cytokines (IFNγ and TNFα) in SW620 cells (S7 Fig), excluding the possibility of de novo target expression as a mechanism for bystander killing. We further determined that proximity between BiTE^®^-activated T cells and bystander cells was required and that bystander lysis occurred after 18–48 hours. Although the precise mechanisms underlying bystander killing are yet to be determined, we demonstrated that lysis of target-negative bystander cells was cytokine-dependent, required contact with BiTE^®^-activated T cells and involved upregulation of ICAM-1 and FAS on TAA-negative cells.

Cell surface expression of FAS and ICAM-1 was upregulated by exposure of SW620 cells to IFNγ and TNFα, as has been shown for other cell types [31, 38–43], presumably increasing their susceptibility to FAS ligand upregulated by BiTE^®^-activated T cells, and their adhesiveness to activated T cells. Because these events take time, bystander lysis would not be observed in short-term microscopic studies. We propose that T cells activated by BiTE^®^ antibody constructs in the presence of target-positive cells release cytokines that diffuse locally and bind to proximal target-negative cells. This does not lead to direct cytotoxic effects but to the upregulation of cell surface molecules including cell adhesion molecule ICAM-1 and death receptor FAS on bystander cells. Expression of these molecules, and likely others yet to be determined, render bystander TAA-negative cells susceptible to killing by T cells even in the absence of a regular cytolytic synapse (Fig 1B). Upregulation of FAS ligand, as observed in BiTE^®^-activated T cells, and of FAS on TAA-negative cells may be a critical element for bystander lysis as supported by the partial protective effect of a blocking anti-FAS antibody.

T cell-produced IFNγ and TNFα were shown to be critical for bystander killing of antigen loss variants in mouse models [44], and studies showing fibroblast lysis in graft-versus-leukemia models suggested that a local pro-inflammatory environment led to upregulation of adhesion molecules on surrounding tissue and granzyme B-mediated apoptosis of bystander cells when in direct contact with T cells [45].

Cytokine-induced upregulation of ICAM-1 on bystander cells likewise may be important for bystander lysis, which is supported by the partially protective effect of a blocking anti-ICAM-1 antibody. A critical role for ICAM-1 binding to LFA-1 on T cells in target cell lysis is well described [11]. The affinity of LFA-1 to ICAM-1 increases after T cell activation [46] and a high-affinity form of LFA-1 is critical for facilitating T cell activation [47]. ICAM-1/LFA-1 interaction provides costimulation in the absence of CD28 [48, 49], indicating that LFA-1 function in T cells is not limited to adherence. Human ICAM-1, when transfected into a mouse melanoma cell line, was reported to function in both cell adhesion and co-stimulation of lymphokine-activated killer (LAK) T cells [50]. TCR-dependent and -independent LFA-1 signaling have been shown to regulate T cell activation and TCR signaling, and key signaling pathways have been identified [32, 51, 52]. We propose that cytokine-induced ICAM-1 on bystander TAA-negative cells can enable interaction with BiTE^®^-activated T cells expressing high-affinity LFA-1. This enhanced interaction may facilitate lysis of TAA-negative cells by FAS/FAS ligand interaction and, perhaps, by delivery of cytolytic granule content via ‘pseudo synapses.’ Lysis of bystanding antigen-negative cells by FAS-FASL, but not by perforin, has been reported to require LFA-1 on the surface of TCR-activated CTLs [53]. Similarly in a papillomavirus model, antigen-free bystander cells were lysed in a FAS- and ICAM-1-dependent fashion, where expression of FASL and LFA-1 on activated T cells was necessary, but neither alone was sufficient for bystander lysis [54].

Consistent with these studies of bystander killing through antigen-specific T cells, our results demonstrate that TAA-negative bystander cells are effectively lysed by BiTE^®^-activated T cells when TAA-positive target cells are present. Inflammatory cytokines, ICAM-1 and FAS play a central role in this localized response.

Although using blinatumomab and B cell malignancy cells in this study may have provided clinical relevance, we expect the mechanism described herein to be primarily important in the solid tumor setting where there would be little opportunity for factors released by activated T cells to diffuse or be diluted; therefore, our studies were focused on EGFR/CD3 and solid tumor cells. Our goal was to use defined cancer cell lines and an EGFR/CD3-bispecific tool BiTE^®^ in order to establish the basic characteristics and mechanism of a bystander killing effect. At this time, we have made a preclinical observation that will need to be tested in solid tumors when such BiTE^®^ antibody constructs are available and approved for clinical evaluation. According to our proposed model, there is little reason to assume that a bystander effect may contribute much to the activity of blinatumomab in ALL. In blood, T cells may not reach the critical density, are subject to shear forces, and newly released cytokines are swiftly diluted. While a bystander effect may occur in bone marrow where cells are densely packed, we currently have no means to study a bystander killing in bone marrow samples of ALL patients treated with blinatumomab.

Clinical use of blinatumomab has provided no data to indicate that blinatumomab-mediated T cell activation is associated with damage to (ICAM-1-expressing) vascular endothelial cells. In patients, blinatumomab administration has been reported to result in redistribution of T cells at the start of each treatment cycle, likely due to increased adhesion of T cells to vascular endothelium [55]. The fact that this adhesion is not associated with bystander killing is possibly due to the requirement for additional steps beyond ICAM-1 expression (e.g., local action of cytokines, activation of FAS/FASL axis) that might not be possible in circulation. The bystander killing mechanism we propose would likely be restricted to solid tumor settings where there would be little opportunity for factors released by activated T cells to diffuse or be diluted. Thus, extratumoral toxicity is expected to be limited because diffusion of T cell-derived cytokines is restricted to nearby bystander cells in solid tumors, and T cell engagement, cytokine release and serial killing by BiTE^®^-activated T cells diminish over time as the TAA-positive population is lysed. This preliminary model for bystander killing observed with an EGFR tool BiTE^®^ will need to be tested when additional BiTE^®^ therapeutics for solid tumors are available for testing.

## Supporting information

S1 Table Cytokine expression increased upon activation of T cells with EGFR BiTE^®^ and EGFR-expressing cells. EGFR-positive NUGC4 and EGFR-negative SW620 cells were treated with EGFR BiTE^®^ and T cells (E:T = 10:1) for 48 hours prior to collecting medium for assays. BiTE^®^-activated T cells were prepared as described in Methods. Soluble factors were measured by ELISA (Granzyme B, FASL) or MSD (IL-6, IL-1β) as described in Methods. Media from four replicate wells of a 96-well plate were combined prior to measurement.
(PDF)

S1 Fig NUGC4 cells were positive and SW620 cells were negative for EGFR expression. (A) Cell surface protein expression was determined on live cells by flow cytometry with anti-EGFR antibody cetuximab (cmab). (B) Binding of cmab-derived anti-EGFR BiTE^®^ or negative control BiTE^®^ (MEC14) was determined by flow cytometry (see Methods). (C) Cell surface EGFR expression level was determined (as antibody binding capacity) by fluorescence quantitation with anti-EGFR antibody and Quantum^™^ Simply Cellular kit (Bangs Laboratories, Inc.) according to manufacturer’s instructions.
(TIF)

S2 Fig EGFR-negative and -positive populations were quantitatively enumerated to assess bystander killing. (A) Representative images of untreated EGFR-positive/EGFR-negative mixed cultures at 48 hours stained with EGFR antibody, with percent EGFR-positive shown at the time of plating (blue = nuclear stain; green = EGFR, scale bar = 30 μm.). (B) Population distribution and fluorescence gating strategy used for analysis in Fig 3. Dotted line represents the threshold for EGFR fluoresence. (C) EGFR-positive NUGC4 cells and EGFR-negative SW620 cells were mixed together in various ratios and incubated with T cells (E:T ratio 10:1) and 0, 1.2 or 11 pM EGFR BiTE^®^ for 48 hours as described for Fig 3. EGFR-positive (left panel) and EGFR-negative (right panel) populations were analyzed as described for Fig 3 (N = 4, mean +/- sd). (D) Luciferase-labeled EGFR-negative AML cells (MOLM13-LUC) were mixed with unlabeled EGFR-positive NUGC4 cells (1:1) and cytotoxicity was measured by luminescence (Steady-Glo^®^, Promega) after a 48-hour incubation with T cells (E:T 10:1) and EGFR BiTE^®^, negative control MEC14 BiTE^®^ or positive control CD33 BiTE^®^. N = 3, mean +/-sd.
(TIF)

S3 Fig HCT116 cells express EGFR and are susceptible to EGFR BiTE^®^-mediated cytotoxicity. (A) HCT116 cells were incubated for 48 hours with EGFR BiTE^®^ and T cells (E:T 10:1). Cytotoxicity was measured by nuclear count with cellular imaging (N = 4, mean +/- sd). (B) Live HCT116 cells were stained with anti-EGFR antibody (ThermoFisher) to confirm EGFR surface expression.
(TIF)

S4 Fig Soluble factors secreted by activated T cells or added exogenously were not directly cytotoxic. EGFR BiTE^®^, T cells and NUGC4 cells (10:1 E:T ratio) were incubated in 96-well plates for 48 hours; supernatants were either (A) transferred directly (transfer medium + T cells) or (B) clarified by centrifugation (transfer medium only) prior to transfer to 96-well plates containing SW620 cells, followed by a 48-hour incubation. (C) SW620 and T cells were cultured with EGFR BiTE^®^ for 48 hours (no transfer control). (D) T cells alone or T cells + EGFR BiTE^®^ + NUGC4 cells were added to the top chamber of Transwell^®^ assays with 1 μm and 5 μm membranes. After 72 hours, T cell counts in the bottom chambers were determined with CellTiter-Glo^®^ (Promega) and percent of T cells transiting each membrane was determined (compared to equal number of T cells placed in the bottom chamber at time of plating). (E) NUGC4 cells and SW620 cells were treated with IFNγ and TNFα alone, or in combination for 24 hours; cell number was determined by nuclear count with an imaging assay. (N = 3, mean +/- sd for all assays).
(TIF)

S5 Fig Representative images demonstrating ICAM-1 and FAS induction by recombinant cytokines and BiTE^®^-activated T cells. Immunofluorescence images for Fig 6 showing ICAM-1 upregulation in NUGC4 cells by (A) 12.5ng/ml each IFNγ + TNFα or (B) 33 pM BiTE^®^ (A, B, blue = nuclear stain, red = ICAM-1 staining). Representative images of SW620 cells showing upregulation of ICAM-1 by (C) 12.5ng/ml each IFNγ + TNFα or (D) BiTE^®^-activated T cells. Representative images of SW620 cells showing upregulation of FAS by (E) 12.5ng/ml each IFNγ + TNFα or (F) BiTE^®^-activated T cells (C-F, blue = nuclear stain, green = ICAM-1 or FAS staining). Scale bar = 30 μm.
(TIF)

S6 Fig FAS agonistic antibody only induces bystander killing after cytokine treatment in SW620 cells. Cytokine-pretreated (left panel) or untreated (right panel) SW620 cells were incubated +/- FAS neutralizing antibody or control antibody for one hour before adding FAS agonistic antibody for 24 hours. Cell count was determined by imaging; N = 6, mean +/- sd. Significance values: ns, P > 0.05; *P < 0.05; **P < 0.01; ***P < 0.001; ****P < 0.0001.
(TIF)

S7 Fig BiTE^®^, IFNγ and TNFα do not induce EGFR expression on SW620 cells. SW620 cells were treated with a dose response of BiTE^®^ (plus T cells, 10:1 E:T) or cytokines for 24 or 48 hours and EGFR expression was assessed by cellular imaging as described. Untreated NUGC4 cells were used as a positive control. For clarity, single doses are shown: 200 pM BiTE^®^ and 5 ng/ml each IFNγ and TNFα. N = 2 (BiTE^®^), 4 (cytokines) or 3 (untreated controls); bar = mean. Representative images demonstrating EGFR staining from which the quantitative data in (A) were derived are shown in B-F. (B) Positive control NUGC4, no treatment; (C) SW620, no treatment; (D) SW620 + 200 pM EGFR BiTE^®^ at 48 hours; (E) SW620 + 5ng each IFNγ and TNFα at 24 hours; (F) SW620 + 5ng each IFNγ and TNFα at 48 hours.
(TIF)
