# Supplementary material for: Bispecific T cell engager (BiTE®) antibody constructs can mediate bystander tumor cell killing
Source: PLoS One. 2017 Aug 24;12(8):e0183390. doi: 10.1371/journal.pone.0183390 (PMC5570333; doi:10.1371/journal.pone.0183390)
Supplement: S1 Table — EGFR-positive NUGC4 and EGFR-negative SW620 cells were treated with EGFR BiTE® and T cells (E:T = 10:1) for 48 hours prior to collecting medium for assays. BiTE®-activated T cells were prepared as described in Methods. Soluble factors were measured by ELISA (Granzyme B, FASL) or MSD (IL-6, IL-1β) as described in Methods. Media from four replicate wells of a 96-well plate were combined prior to measurement. (PDF) [file pone.0183390.s001.pdf]

S1 Table. FASL, Granzyme B, IL-6 and IL-1β expression increase upon activation of T cells with EGFR BiTE® and EGFR-expressing cells.

Cells (EGFR +/-)	T cells	EGFR BiTE® (pM)	ELISA		MSD	
			FASL (pg/ml)	GZMB (pg/ml)	IL-6 (pg/ml)	IL-1β (pg/ml)
NUGC4 (+)	none	0	<1	<50	<2	<2
NUGC4 (+)	resting	0	2.504	<50	<2	<2
NUGC4 (+)	resting	4	61.81	>1000	3.500	<2
NUGC4 (+)	resting	11	80.05	>1000	17.60	7.600
SW620 (-)	none	0	<1	<50	<2	<2
SW620 (-)	resting	0	1.937	<50	<2	<2
SW620 (-)	resting	4	1.910	<50	<2	<2
SW620 (-)	resting	11	3.304	<50	<2	<2
NUGC4 (+)	BiTE®-activated	0	76.85	>1000	<2	<2
NUGC4 (+)	BiTE®-activated	4	242.4	>1000	3.800	<2
NUGC4 (+)	BiTE®-activated	11	368.1	>1000	12.80	5.600
SW620 (-)	BiTE®-activated	0	57.59	>1000	<2	<2
SW620 (-)	BiTE®-activated	4	55.47	>1000	<2	<2
SW620 (-)	BiTE®-activated	11	59.69	>1000	<2	<2
Assay Range (pg/ml)			1 - 1000	50 - 1000	2 - 10000	2 - 10000
