# Supplementary figures and images for: Bispecific T cell engager (BiTE®) antibody constructs can mediate bystander tumor cell killing
Source: PLoS One. 2017 Aug 24;12(8):e0183390. doi: 10.1371/journal.pone.0183390 (PMC5570333; doi:10.1371/journal.pone.0183390)

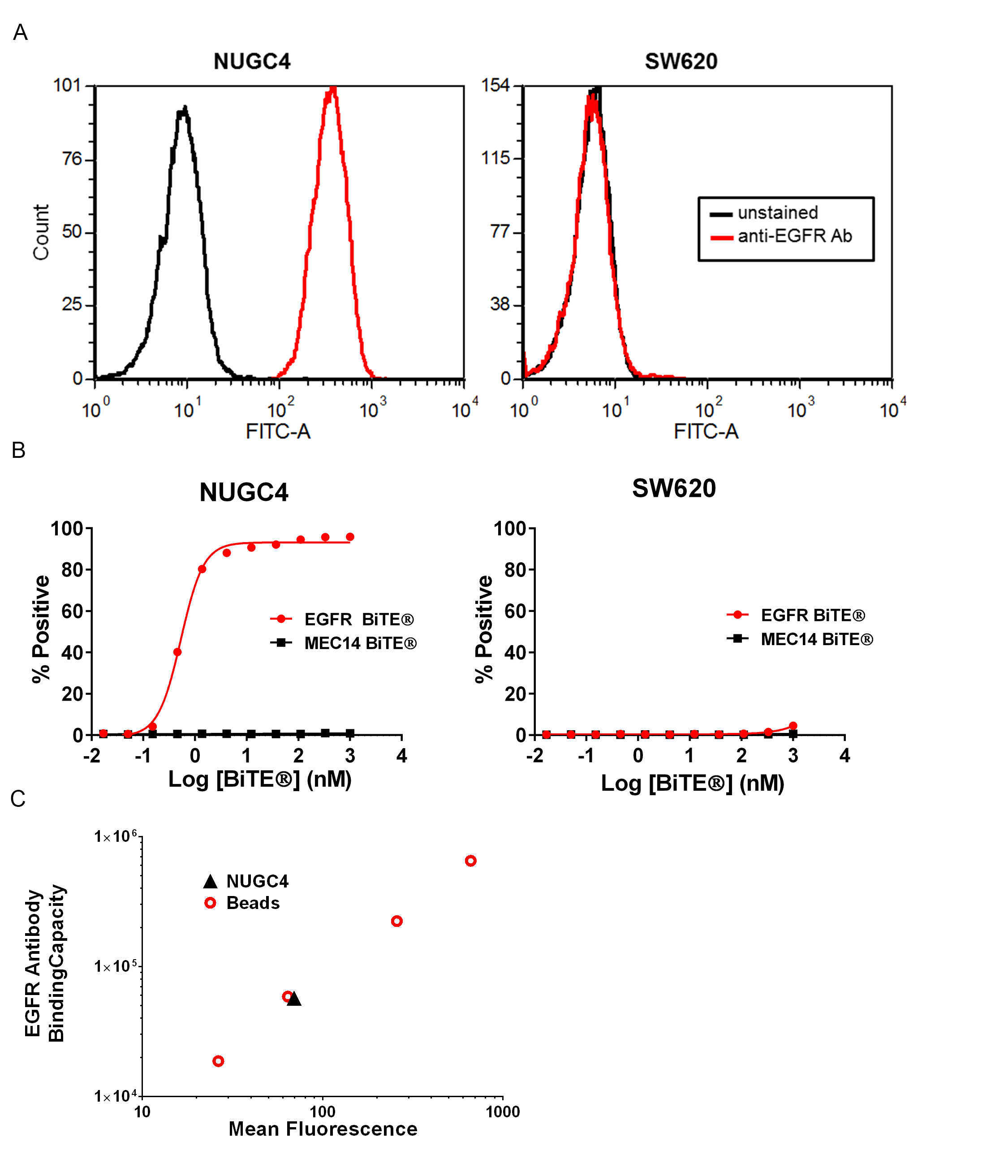

Supplement: S1 Fig — (A) Cell surface protein expression was determined on live cells by flow cytometry with anti-EGFR antibody cetuximab (cmab). (B) Binding of cmab-derived anti-EGFR BiTE® or negative control BiTE® (MEC14) was determined by flow cytometry (see Methods). (C) Cell surface EGFR expression level was determined (as antibody binding capacity) by fluorescence quantitation with anti-EGFR antibody and Quantum™ Simply Cellular kit (Bangs Laboratories, Inc.) according to manufacturer’s instructions. (TIF) [file pone.0183390.s002.tif]

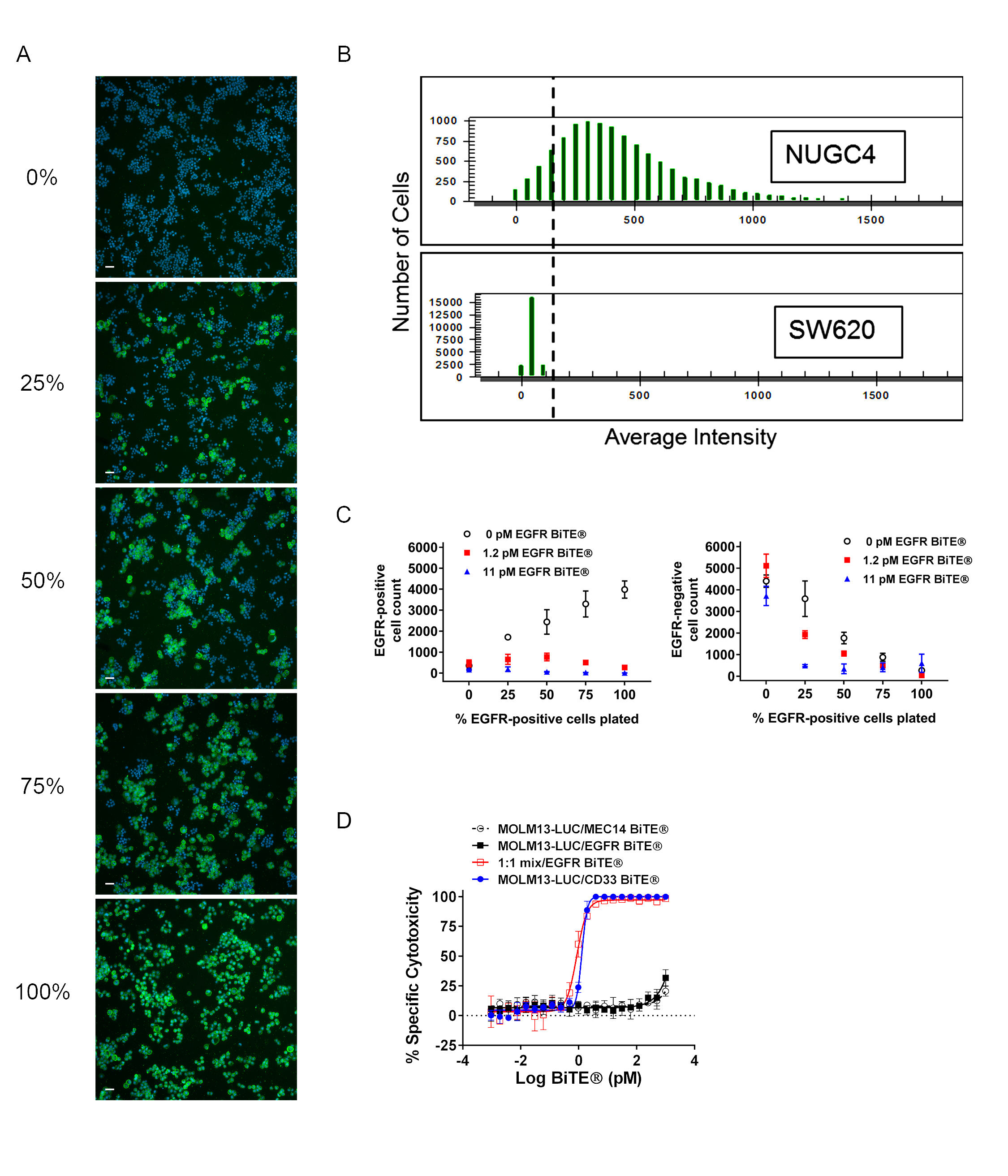

Supplement: S2 Fig — (A) Representative images of untreated EGFR-positive/EGFR-negative mixed cultures at 48 hours stained with EGFR antibody, with percent EGFR-positive shown at the time of plating (blue = nuclear stain; green = EGFR, scale bar = 30 μm.). (B) Population distribution and fluorescence gating strategy used for analysis in Fig 3. Dotted line represents the threshold for EGFR fluoresence. (C) EGFR-positive NUGC4 cells and EGFR-negative SW620 cells were mixed together in various ratios and incubated with T cells (E:T ratio 10:1) and 0, 1.2 or 11 pM EGFR BiTE® for 48 hours as described for Fig 3. EGFR-positive (left panel) and EGFR-negative (right panel) populations were analyzed as described for Fig 3 (N = 4, mean +/- sd). (D) Luciferase-labeled EGFR-negative AML cells (MOLM13-LUC) were mixed with unlabeled EGFR-positive NUGC4 cells (1:1) and cytotoxicity was measured by luminescence (Steady-Glo®, Promega) after a 48-hour incubation with T cells (E:T 10:1) and EGFR BiTE®, negative control MEC14 BiTE® or positive control CD33 BiTE®. N = 3, mean +/-sd. (TIF) [file pone.0183390.s003.tif]

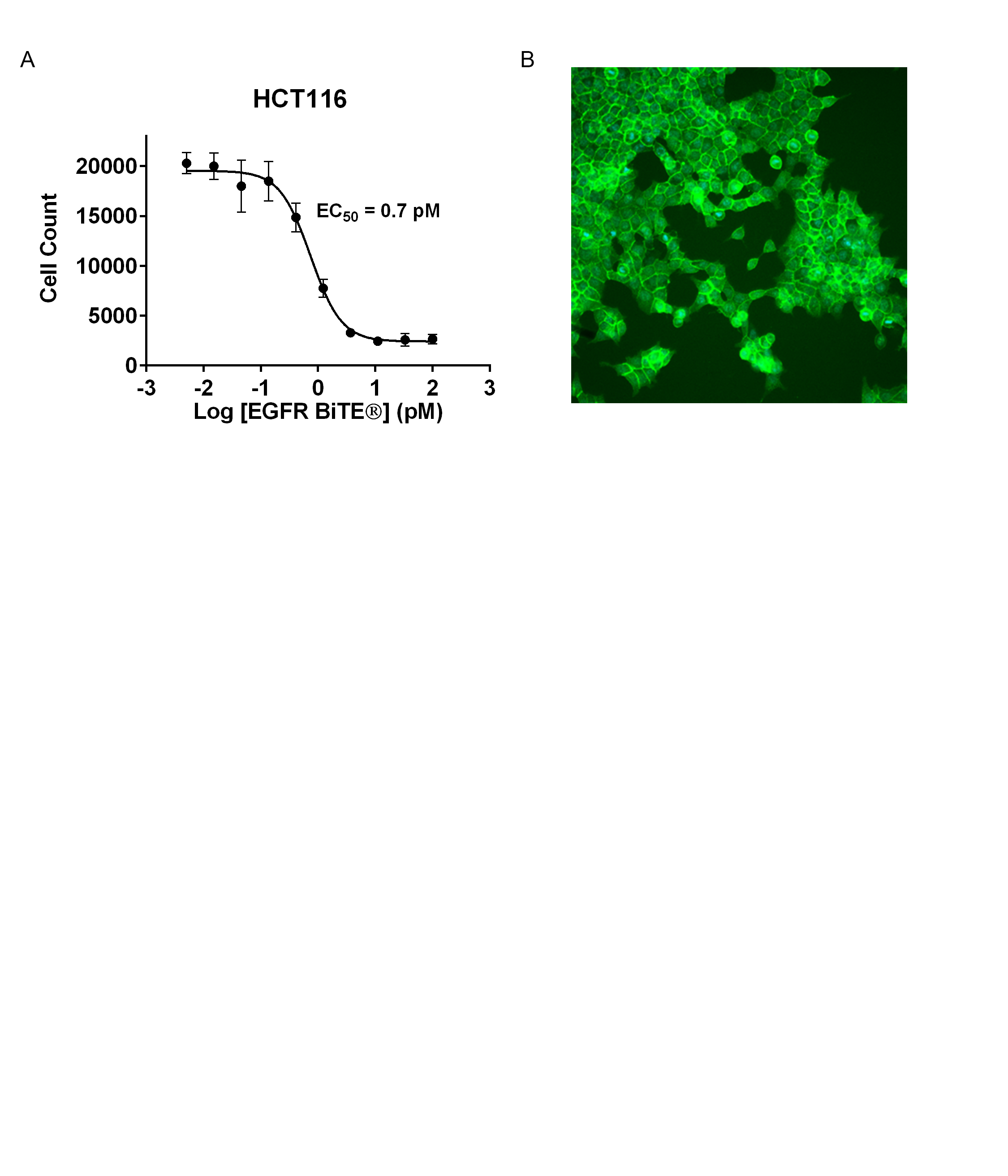

Supplement: S3 Fig — (A) HCT116 cells were incubated for 48 hours with EGFR BiTE® and T cells (E:T 10:1). Cytotoxicity was measured by nuclear count with cellular imaging (N = 4, mean +/- sd). (B) Live HCT116 cells were stained with anti-EGFR antibody (ThermoFisher) to confirm EGFR surface expression. (TIF) [file pone.0183390.s004.tif]

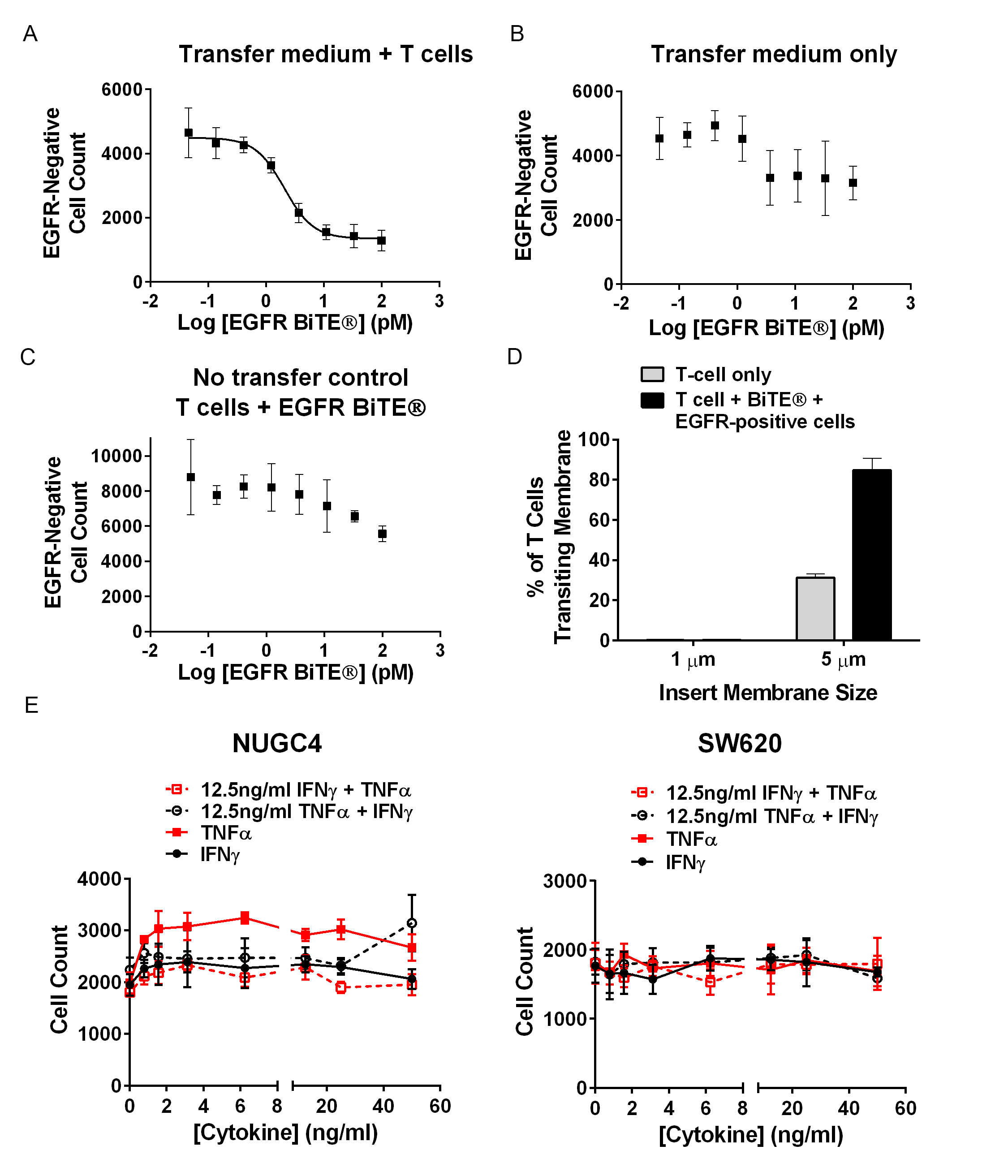

Supplement: S4 Fig — EGFR BiTE®, T cells and NUGC4 cells (10:1 E:T ratio) were incubated in 96-well plates for 48 hours; supernatants were either (A) transferred directly (transfer medium + T cells) or (B) clarified by centrifugation (transfer medium only) prior to transfer to 96-well plates containing SW620 cells, followed by a 48-hour incubation. (C) SW620 and T cells were cultured with EGFR BiTE® for 48 hours (no transfer control). (D) T cells alone or T cells + EGFR BiTE® + NUGC4 cells were added to the top chamber of Transwell® assays with 1 μm and 5 μm membranes. After 72 hours, T cell counts in the bottom chambers were determined with CellTiter-Glo® (Promega) and percent of T cells transiting each membrane was determined (compared to equal number of T cells placed in the bottom chamber at time of plating). (E) NUGC4 cells and SW620 cells were treated with IFNγ and TNFα alone, or in combination for 24 hours; cell number was determined by nuclear count with an imaging assay. (N = 3, mean +/- sd for all assays). (TIF) [file pone.0183390.s005.tif]

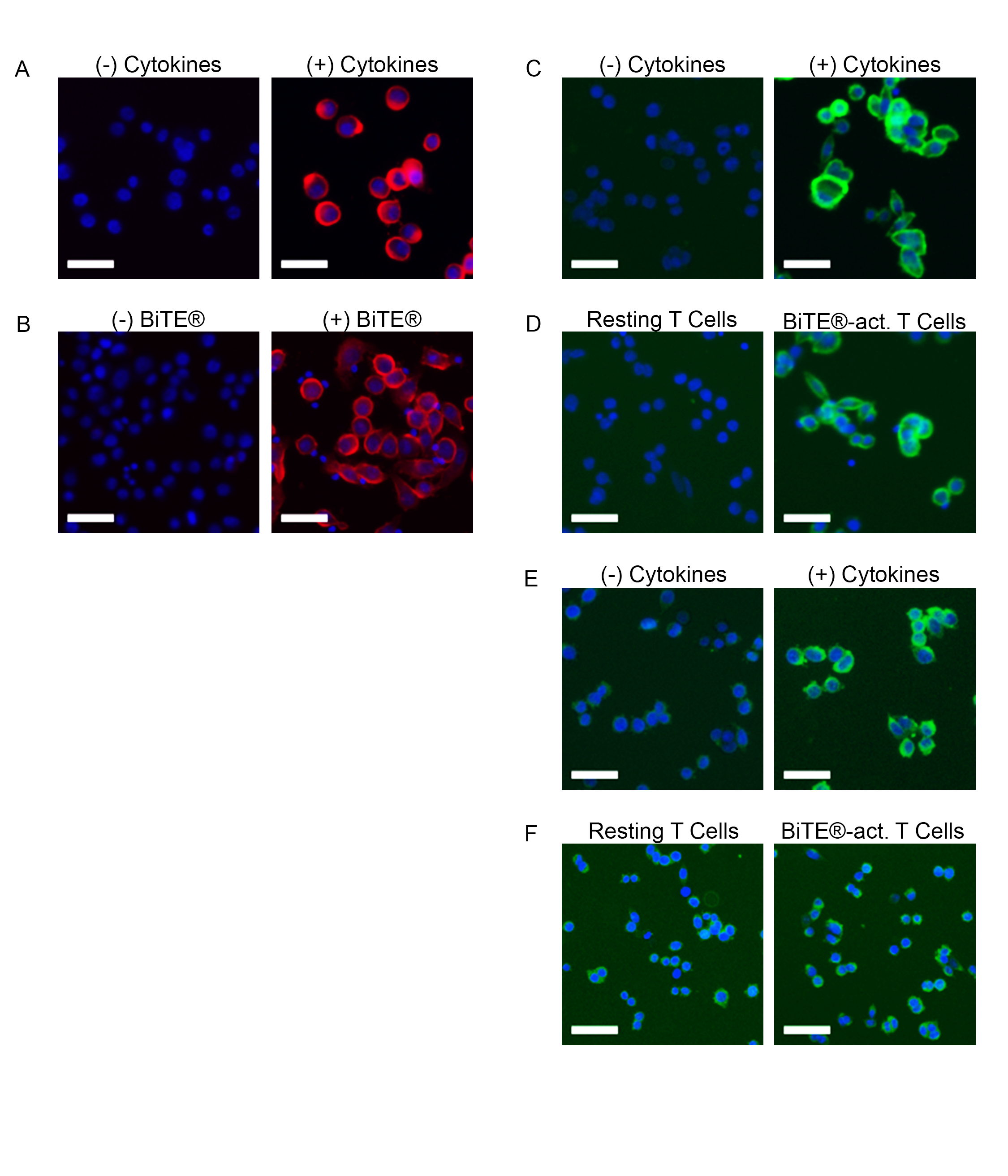

Supplement: S5 Fig — Immunofluorescence images for Fig 6 showing ICAM-1 upregulation in NUGC4 cells by (A) 12.5ng/ml each IFNγ + TNFα or (B) 33 pM BiTE® (A, B, blue = nuclear stain, red = ICAM-1 staining). Representative images of SW620 cells showing upregulation of ICAM-1 by (C) 12.5ng/ml each IFNγ + TNFα or (D) BiTE®-activated T cells. Representative images of SW620 cells showing upregulation of FAS by (E) 12.5ng/ml each IFNγ + TNFα or (F) BiTE®-activated T cells (C-F, blue = nuclear stain, green = ICAM-1 or FAS staining). Scale bar = 30 μm. (TIF) [file pone.0183390.s006.tif]

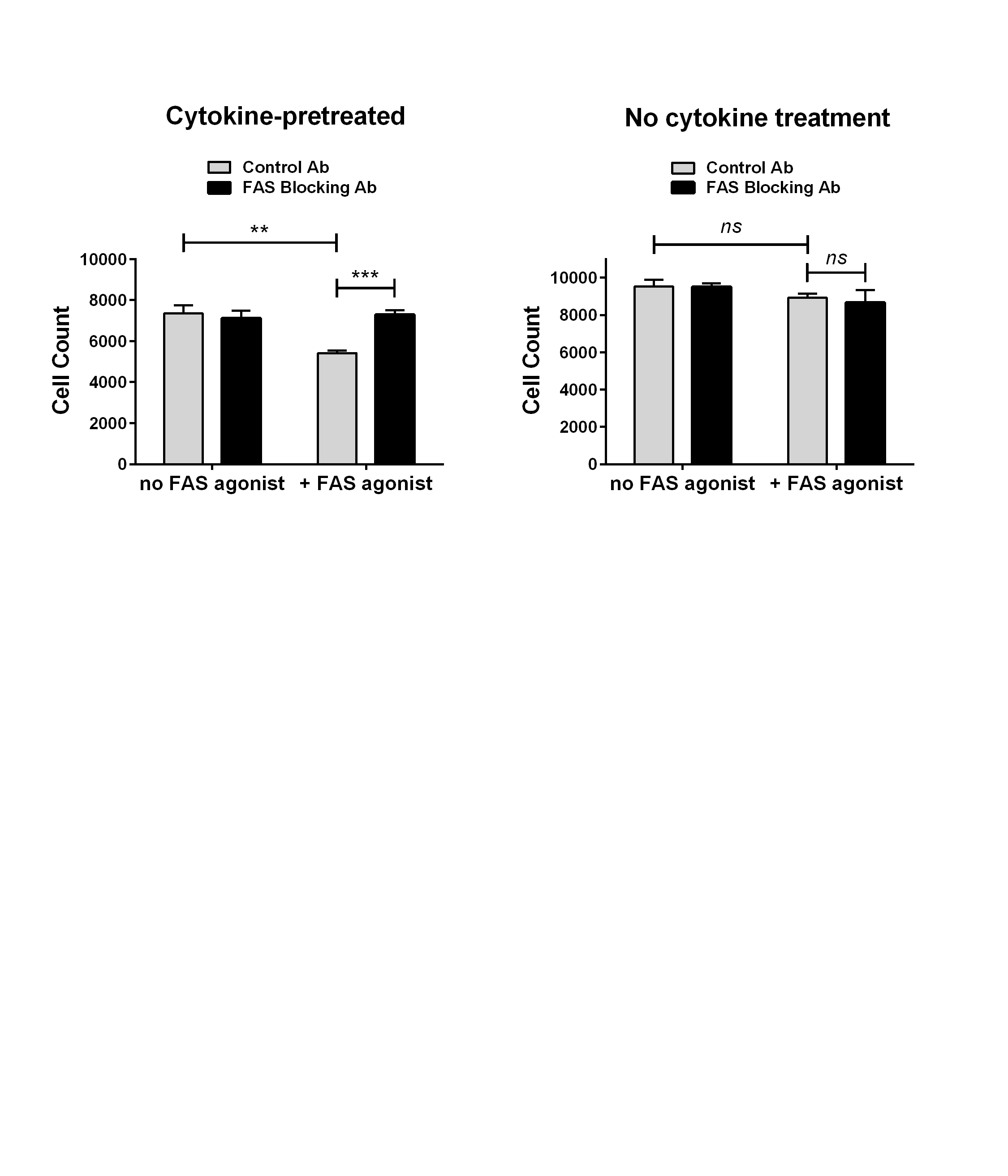

Supplement: S6 Fig — Cytokine-pretreated (left panel) or untreated (right panel) SW620 cells were incubated +/- FAS neutralizing antibody or control antibody for one hour before adding FAS agonistic antibody for 24 hours. Cell count was determined by imaging; N = 6, mean +/- sd. Significance values: ns, P > 0.05; *P < 0.05; **P < 0.01; ***P < 0.001; ****P < 0.0001. (TIF) [file pone.0183390.s007.tif]

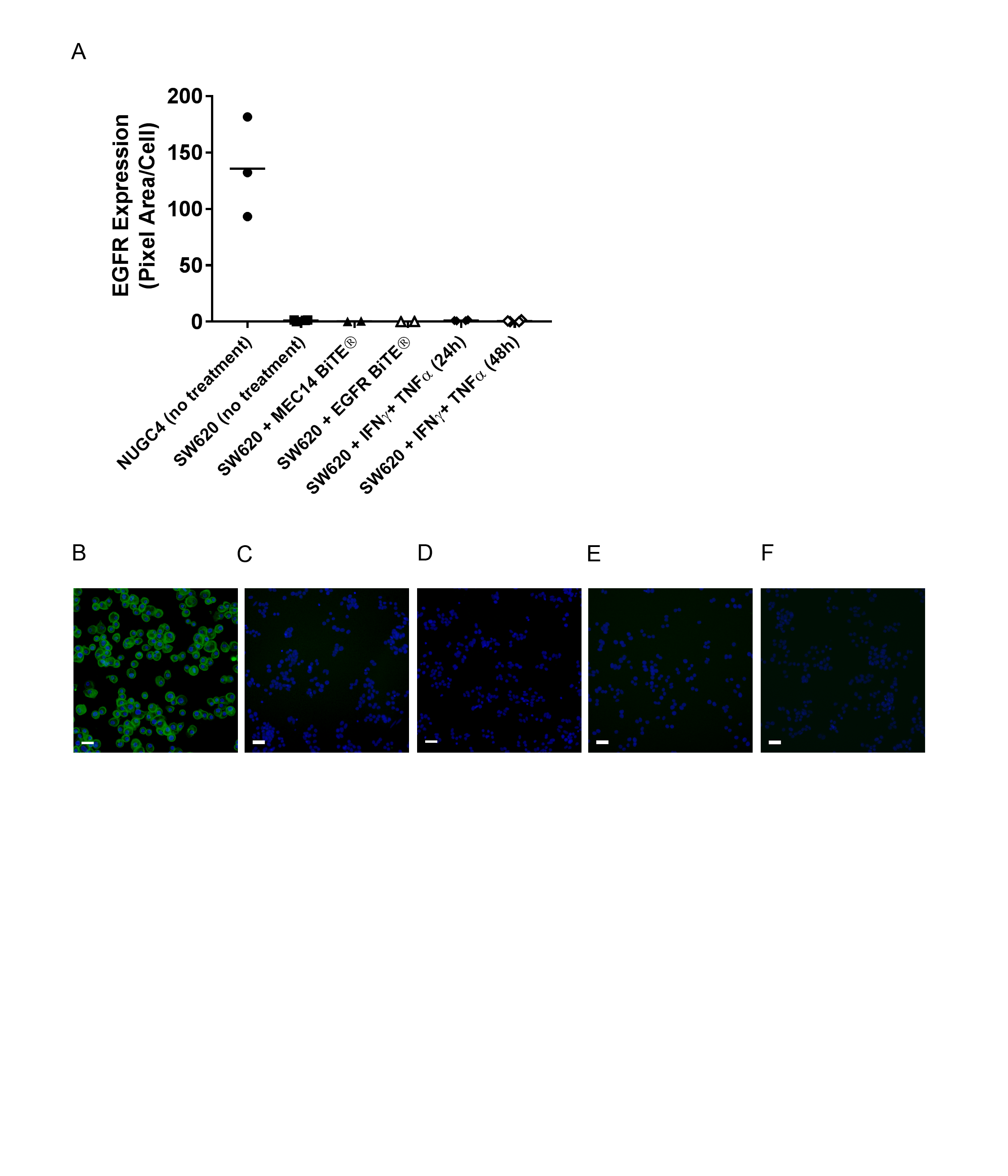

Supplement: S7 Fig — SW620 cells were treated with a dose response of BiTE® (plus T cells, 10:1 E:T) or cytokines for 24 or 48 hours and EGFR expression was assessed by cellular imaging as described. Untreated NUGC4 cells were used as a positive control. For clarity, single doses are shown: 200 pM BiTE® and 5 ng/ml each IFNγ and TNFα. N = 2 (BiTE®), 4 (cytokines) or 3 (untreated controls); bar = mean. Representative images demonstrating EGFR staining from which the quantitative data in (A) were derived are shown in B-F. (B) Positive control NUGC4, no treatment; (C) SW620, no treatment; (D) SW620 + 200 pM EGFR BiTE® at 48 hours; (E) SW620 + 5ng each IFNγ and TNFα at 24 hours; (F) SW620 + 5ng each IFNγ and TNFα at 48 hours. (TIF) [file pone.0183390.s008.tif]
